# Magnetic Nanoparticle-Based Hyperthermia Mediates Drug Delivery and Impairs the Tumorigenic Capacity of Quiescent Colorectal Cancer Stem Cells

**DOI:** 10.1021/acsami.0c21349

**Published:** 2021-04-02

**Authors:** Soraia Fernandes, Tamara Fernandez, Sabrina Metze, Preethi B. Balakrishnan, Binh T. Mai, John Conteh, Claudia De Mei, Alice Turdo, Simone Di Franco, Giorgio Stassi, Matilde Todaro, Teresa Pellegrino

**Affiliations:** † Istituto Italiano di Tecnologia (IIT), via Morego 30, 16163 Genova, Italy; ‡ PROMISE Department,Piazza delle Cliniche 2, University of Palermo, 90133 Palermo, Italy; § DICHIRONS Department, University of Palermo, Via del Vespro 129, 90133 Palermo, Italy

**Keywords:** magnetic hyperthermia, magnetic nanoparticles, doxorubicin, cancer stem cells, colorectal cancer

## Abstract

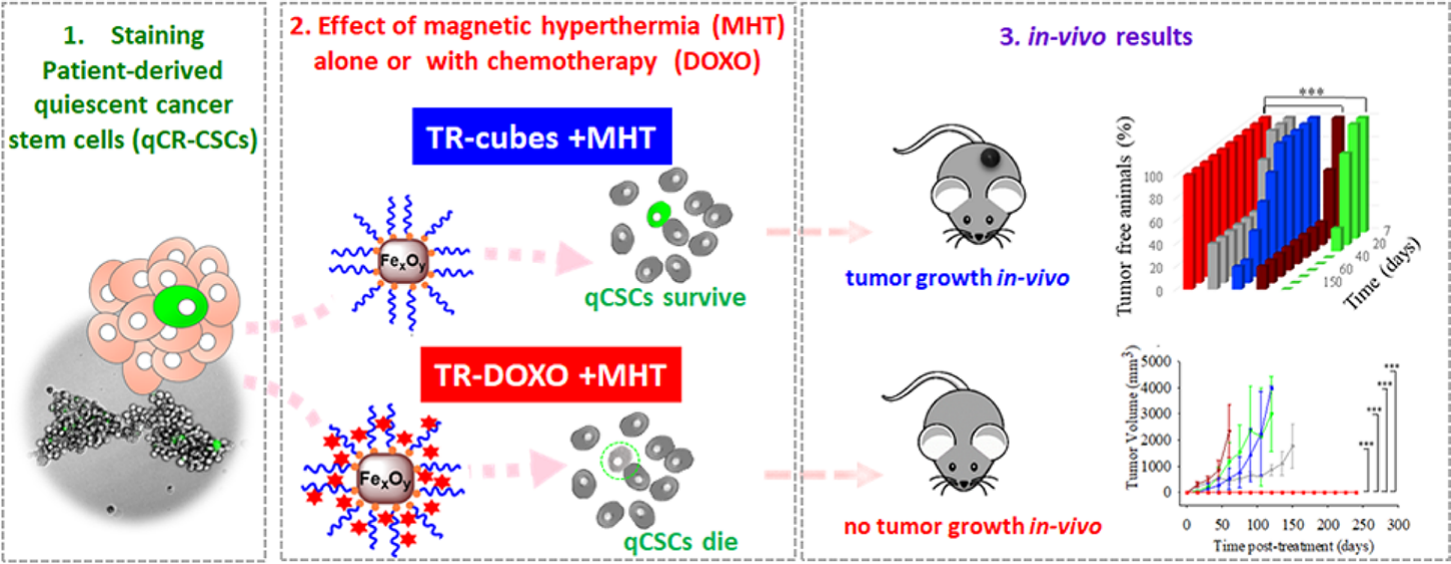

Cancer stem cells (CSCs) are the tumor cell subpopulation responsible for resistance to chemotherapy, tumor recurrence, and metastasis. An efficient therapy must act on low proliferating quiescent-CSCs (q-CSCs). We here investigate the effect of magnetic hyperthermia (MHT) in combination with local chemotherapy as a dual therapy to inhibit patient-derived colorectal qCR-CSCs. We apply iron oxide nanocubes as MHT heat mediators, coated with a thermoresponsive polymer (TR-Cubes) and loaded with DOXO (TR-DOXO) as a chemotherapeutic agent. The thermoresponsive polymer releases DOXO only at a temperature above 44 °C. In colony-forming assays, the cells exposed to TR-Cubes with MHT reveal that qCR-CSCs struggle to survive the heat damage and, with a due delay, restart the division of dormant cells. The eradication of qCR-CSCs with a complete stop of the colony formation was achieved only with TR-DOXO when exposed to MHT. The *in vivo* tumor formation study confirms the combined effects of MHT with heat-mediated drug release: only the group of animals that received the CR-CSCs pretreated, *in vitro*, with TR-DOXO and MHT lacked the formation of tumor even after several months. For DOXO-resistant CR-CSCs cells, the same results were shown, *in vitro,* when choosing the drug oxaliplatin rather than DOXO and applying MHT. These findings emphasize the potential of our nanoplatforms as an effective patient-personalized cancer treatment against qCR-CSCs.

## Introduction

Tumor heterogeneity is a major obstacle for the successful treatment of solid cancers.^1^ CSCs have been identified as the subpopulation of tumor mass able to self-renew through asymmetric divisions, providing fast proliferating cells and quiescent cells. The latter ones are able to repopulate and re-establish an entire tumor mass starting from one single cell under stress-related conditions.^2−6^ Therefore, the existence of this subset of tumor cells significantly contributes to tumor heterogeneity with consequent resistance to chemo- or radiotherapy.^5,7,8^ Hence, many efforts have been dedicated to identify and isolate CSCs from different tumors, including colorectal (CR) cancer, the third most common cancer worldwide, to study their survival to antitumoral treatment and to design more efficacious targeted therapies for their eradication.^5,6,9^ In particular, when using antiproliferating agents such as chemotherapeutic agents or radiation therapy, it was found that reduced cell cycling and increased DNA repairing capacity are typical resistance mechanisms of q-CSCs that make them less susceptible to therapy than the bulk cancer cells.^10,11^ Several studies have shown that by eradicating the fast proliferating tumor cells while sparing q-CSCs, the disease’s progression is only temporarily suppressed. In fact, killing the proliferating cells stimulates the reawakening of q-CSCs that have evaded therapy, eventually leading to cancer relapse.^3,10^

Among all the different therapeutic approaches for treating cancer, hyperthermia employs a moderate temperature (ca. 41–46 °C) to damage tumor cells.^12^ It is currently used in clinics as an adjuvant treatment to the more common types of antitumoral therapies.^13^ Indeed, if applied simultaneously or shortly after radiotherapy or chemotherapy, hyperthermia can interfere with the repair of therapy-induced DNA damage, thereby contributing to kill tumor cells.^14−16^ Novel nanoparticle-based platforms are emerging as a promising new technology to deposit heat in a more localized and selective mode enabling, simultaneously, the remote activation of the nanoparticles working as heat transducers. In MHT, the heat is produced by magnetic nanoparticles exposed to an alternating magnetic field (AMF) at a radiofrequency range (100 kHz) that has no body penetration restrictions and is not harmful for human patients. MHT has been introduced in clinics for the treatment of glioblastoma and is now under clinical trials for prostate and metastatic bone cancers.^17,18^ While many works have optimized the use of nanoparticles for cancer treatment, less is reported on the effect of nanoparticle-based hyperthermia on CSC eradication. In photothermal therapy, when using an infrared (IR) source to activate nanoparticles (such as carbon nanotubes^19^ or gold nanoparticles^20^) and generate heat, clear indications of CSCs’ sensibility to heat were observed through viability studies, heat-shock protein (HSP) expression, and DNA damage analysis. However, low tissue penetration depth is a major drawback of light sources used in photothermal treatment.

Sadhukha *et al*. demonstrated for the first time the *in vivo* potential of using superparamagnetic iron oxide nanoparticles (SPIONs) as MHT agents to reduce the tumorigenic potential of CSCs from immortalized adenocarcinoma cell lines.^21^ More recently, Kwon and co-workers have reported the use of magnetic nanoclusters to demonstrate the cytotoxic effect *in vitro* of MHT treatment on CSCs using a breast cancer cell line.^22^ While this study is pioneering the field of MHT on CSCs, it employs AMF field conditions (*f* = 290 kHz; *H* = 60 kA/m) that are not suitable for the safe application of MHT therapy in clinics (the product *Hf* should not exceed 5 × 10^9^ Am^–1^ s^–1^ to avoid patient discomfort due to eddy currents at the patient tissue).^23^ The effects of the MHT on CSCs urge to be conducted and evaluated under clinically applied conditions (100 kHz and 24 kA/m) or at an *Hf* factor that is clinically safe.^23^ This requires the use of magnetic nanoparticles with outstanding MHT heat properties.

In a more advanced study, Liu *et al*. exploited the CD20 antibody as a molecular target to accumulate silica-based magnetic nanoparticles on CSCs in a human lung cancer model.^24^ Furthermore, multiple cycles of MHT (up to 10 cycles) under clinically acceptable magnetic field conditions in combination with chemotherapy were performed to effectively reduce the tumor growth *in vivo*.

Recently, we have developed a magnetic nanoparticle-based platform consisting of cubic shape iron oxide nanoparticles (IONCs) coated with a thermoresponsive polymer (TR-Cubes) that can carry and release doxorubicin (TR-DOXO).^25^ Using such TR-DOXO, the release of DOXO is achieved following a heat stimulus provided by the nanocubes under MHT. Our group proved that the DOXO released from the TR-DOXO is significantly accelerated under clinically acceptable AMF conditions. The synergic therapeutic effects of the heat-mediated chemotherapy and the direct heat damage caused a complete eradication of human adenocarcinoma tumors in a xenograft mice model only when the animals were treated with the TR-DOXO and exposed to clinically MHT conditions.^25^ Remarkably, the dose of DOXO needed for the combination therapy was much lower than that used in other *in vivo* studies.^26,27^ This might eventually reduce the side effects of standard chemotherapy doses.

Herein, by exploiting this innovative magnetic nanoplatform (TR-DOXO), we aim to understand the effect of MHT in combination with heat-mediated chemotherapeutic drug release not only on the whole tumor but on a unique dormant cancer subset, the quiescent CSCs. For this purpose, we performed our experiments on CSCs, growing as spheroids, isolated from CR cancer specimens since their resistance to chemotherapeutic treatments is by far more aggressive than that of immortalized tumor cells. More specifically, we have implemented standard assays for identifying qCR-CSCs and study the effects of the MHT with TR-Cubes and TR-DOXO on those cells. Through a variety of *in vitro* analyses including colony-forming assay after fluorescence-activated cell sorting (FACS), confocal and transmission electron microcopy analysis of tumor cells, viability assays such as trypan blue, and apoptotic/necrotic cell quantification by flow cytometry analysis, our study proves the synergistic effects of the dual therapy (MHT and chemotherapy) to completely stop tumor growth. The use of MHT only or drug chemotherapy (DOXO) treatment alone has an effect on the overall viability of tumor spheroids, but it does not fully eradicate the qCR-CSCs as they reawake and regrow the tumor mass both *in vitro* and *in vivo*. Instead, when the cells are treated with TR-DOXO and exposed to MHT, this dual therapy enables the complete eradication of the tumor cells. Moreover, by checking over time the evolution of the number of alive PKH^pos^ qCR-CSCs, after MHT, we have found that the heat stress leads the qCR-CSCs to exit quiescence and start to divide to react and repopulate the tumor colony. At this stage, the DOXO released by the nanocubes is favorably internalized by the dividing CSCs, inducing an additional cytotoxic drug action on the tumor cells. These data were also supported by *in vivo* studies revealing the absence of initiation and relapse potential for the cells previously exposed *in vitro* to MHT with TR-DOXO.

## Results and Discussion

### Characterization and Isolation of Quiescent PKH^pos^ CR-CSCs

Spheroids derived from CR cancer patient tissues (hereafter referred to as CR-CSCs) were isolated as described by Todaro *et al*.^28,29^ CR-CSCs are characterized by the expression of different stemness markers, including CD133 and CD44v6, and by their *in vivo* tumorigenic potential.^28,29^

**Figure 1:**
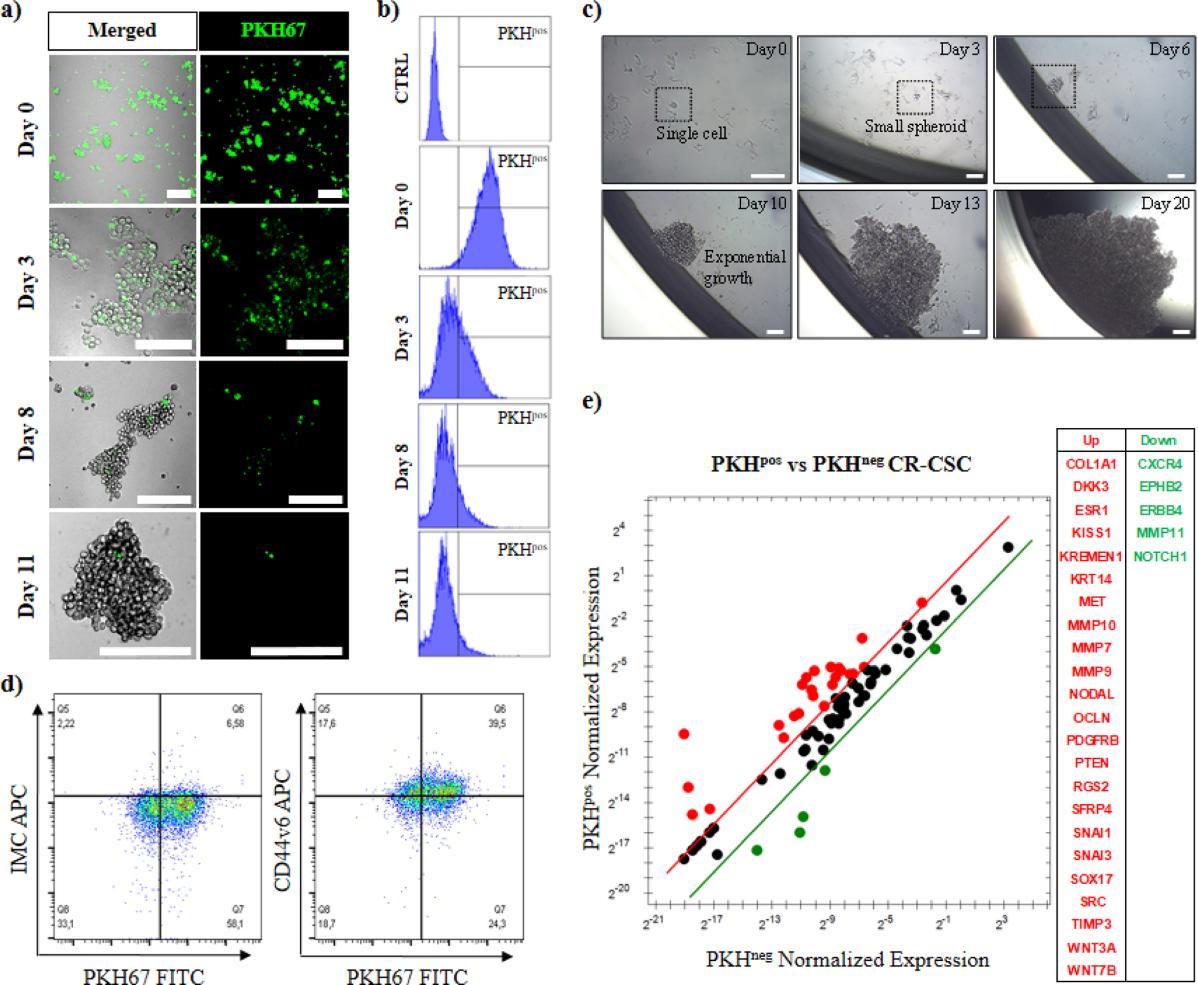
Identification of quiescent colorectal cancer stem cells (PKH^pos^ qCR-CSCs #21). Proliferative PKH67 staining assay enables to identify, after 11 days, the qCR-CSCs by (a) confocal microscopy (the green cells) and (b) flow cytometry analysis. The *y* axis of the histograms represents the counts, while the *x* axis represents the fluorescent signal measured for green PKH67. (c) Self-renewal capability of PKH^pos^ qCR-CSC sorted by FACS and cultured in an isolated environment (one cell per well, scale bars: 50 μm). (d) Flow cytometry analysis of CD44v6 in PKH67-stained CR-CSC (#21) after 11 days of PKH67 staining. (e) Stemness-related gene expression analysis of up- and downregulated genes in FACS-sorted PKH^pos^ and PKH^neg^ CR-CSCs following 11 days of PKH67 staining. Genes showing a more than threefold change in PKH^posg^ than PKH^neg^ CR-CSCs are shown in red and green, respectively.

To study the effects of MHT using TR-Cubes or TR-DOXO on the qCR-CSC spheroid population, we needed to discriminate the qCR-CSCs within the spheroids. A diluting dye proliferative assay based on the use of the membrane green fluorescent PKH67 dye, adapted from a protocol reported for primary breast CSCs, was implemented.^30^ The concept here is to exploit the difference in fluorescent intensity between the two cell populations after the initial staining procedure. The highly proliferative tumor cells were diluting PKH67 in a faster rate, leading to lower to no fluorescence (10–11 days) compared to the slow-dividing qCR-CSCs that retained a strong fluorescent signal for a prolonged period of time as observed by confocal microscopy (Figure 1a). This result was also corroborated by flow cytometry: nearly 10% of green stained cells, representing the qCR-CSCs (PKH^pos^), were found 11 days post-staining (Figure 1b). The self-renewal ability of the sorted PKH^pos^ cells in respect to the nonstained fraction containing the fast proliferating tumor cells (PKH^neg^) was evaluated *in vitro* through a spheroid-forming assay. Plating a single sorted PKH^pos^ qCR-CSC in a 96-well plate led to the formation of small spheroids by day 3, after which they continued to grow, developing a larger cell mass between day 6 and 2 weeks post-seeding, as observed under an optical microscope (Figure 1c). Contrarily, in PKH^neg^ cells, colony formation from a single cell was not observed. In a comparative study, the colony formation assay was slightly modified: after FACS sorting, the PKH^pos^ and PKH^neg^ cells were separately seeded at low cell density (1000 PKH^neg^ cells/well or 100 PKH^pos^ cells/well) and the colony formation was monitored over time (Figure S1). The results confirmed the presence of PKH^pos^ spheroids already after 2 days of culture, indicating a higher self-renewal capacity. On the contrary, PKH^neg^ cells show a much slower growth rate with no signs of sphere formation even after 7 days of culture. Such outcome is in line with data from the literature reporting that fast proliferating cells eventually become exhausted after dividing.^31^ To further verify if the sorted PKH^pos^ cells were enriched in CSCs, we evaluated the expression of CD44v6 as a marker of metastatic cells^29^ (Figure 1d). In accordance with functional studies, PKH^pos^ CR-CSCs displayed higher levels of CD44v6 than the PKH^neg^ cells (Figure 1d). Likewise, PKH^pos^ CR-CSCs showed a more than threefold upregulation in the expression of several crucial stemness genes with respect to fast proliferating PKH^neg^ cells (Figure 1e and Figure S2).

### TR-Cubes and TR-DOXO Preparation and Characterization for the Treatment of CR-CSCs

**Figure 2:**
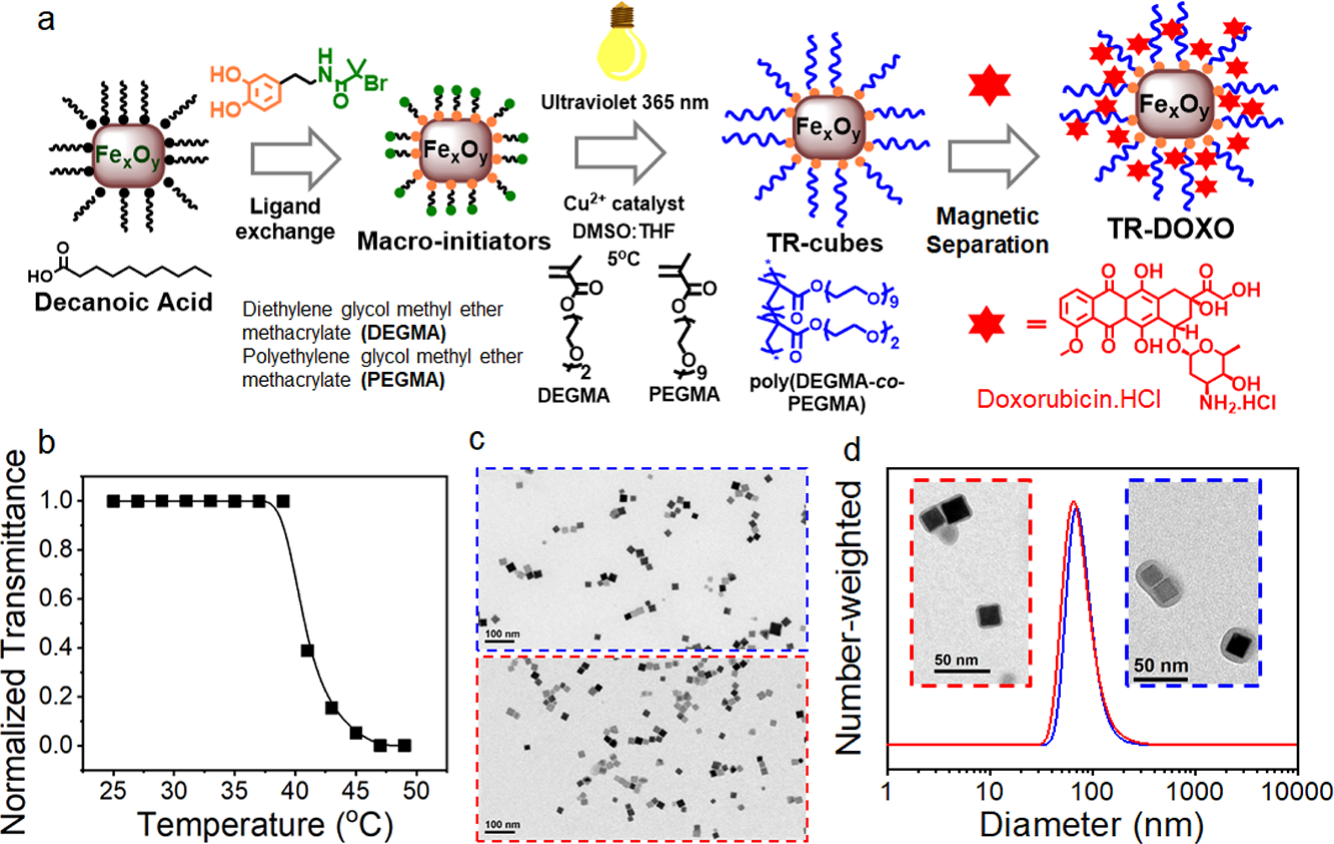
Preparation and characterization of TR-Cubes and TR-DOXO. (a) Scheme of the preparation of TR-Cubes by photoinduced atom transfer radical polymerization and DOXO loading process. IONCs (18 ± 2 nm) first underwent ligand exchange with catechol functionalized ATRP initiators (green–orange molecule) to introduce polymerization initiating sites directly on the nanocube surface. In the second step, monomers and copper catalyst addition under exposure to a UV lamp induce the formation of a P(DEGMA-co-PEGMA) thermoresponsive polymer shell after only 3 h reaction. (b) Transmittance vs temperature curve of TR-Cubes used in this study, resulting in an LCST of 43 °C. (c) TEM image of TR-Cubes (blue frame) and TR-DOXO (red frame). (d) Hydrodynamic size by number for TR-Cubes (blue curve) and TR-DOXO (red curve). Insets of the TEM image of TR-Cubes (blue frame) and TR-DOXO (red frame) showing the polymer shell.

Iron oxide nanocubes (IONCs) functionalized with a thermoresponsive polymer (TR-Cubes), namely, poly(diethylene glycol methyl ether methacrylate-co-polyethylene glycol methyl ether methacrylate) (P(DEGMA-co-PEGMA)), were used as a smart nanocarrier to combine controlled drug delivery and MHT. The TR-Cubes were synthesized by the surface-initiated photoinduced atom transfer radical polymerization (Photo-ATRP) following a recently published protocol of our group (see the scheme of the synthesis in Figure 2a). Initially, IONCs (18 nm) underwent ligand exchange with catechol functionalized ATRP initiators (DOPA-BiBAm) to introduce initiating sites on their surface. Afterward, the solution containing the modified ATRP initiator, the polymer monomers, and the copper catalyst was exposed to a commercial UV lamp (excitation at 365 nm) for a short period (3 h) to induce the formation of P(DEGMA-co-PEGMA) on IONCs’ surface. Recovery of TR-Cubes was obtained by precipitation in diethyl ether followed by ultracentrifugation in a sucrose gradient to remove the nongrafted polymer. Here, Photo-ATRP offers the peculiarity to grow a thick and uniform shell of polymer on the surface of IONCs in a quick manner, thus preventing the IONCs’ tendency to cluster. This feature results in the formation of individual IONCs coated with P(DEGMA-co-PEGMA) that is crucial to maintain their heating performance under clinically relevant MHT. Indeed, a SAR value as high as 250 W·g^–1^ (20 kA·m^–1^, 110 kHz) was determined by calorimetric measurement. Additionally, as shown in Figure 2c, the TR-Cubes deposited from water exhibit a well-dispersed feature on TEM grid, indicating a high stability in aqueous media. In addition, the DLS trace of TR-Cubes in saline reveals a single peak at 86 nm with no sign of aggregation, thus being in good agreement with the TEM result (Figure 2d, blue curve). Notably, a thick and uniform polymer shell surrounding IONCs can be identified at high-magnification TEM, which further confirms a successful surface-initiated polymerization step. Finally, by transmittance measurements, a transition temperature (lower critical solution temperature (LCST)) of 43 °C was determined for the TR-Cubes (Figure 2b).

Doxorubicin hydrochloride (DOXO) was loaded onto TR-Cubes by simple mixing of the drug and the nanocubes in a saline solution at room temperature for an overnight incubation. TR-DOXO were recovered and separated by free DOXO by means of magnetic collection, and the amount of loaded DOXO in the nanocubes was found to be of 40 μg/mg of Fe as determined by DMSO release, as previously described.^25^ Even the TR-DOXO show no sign of aggregation and very good stability in the solution (Figure 2c,d). Interestingly, 30% of loaded DOXO can be released within 1.5 h under MHT when a temperature of 45 °C is reached (110 kHz and 24 kAm^–1^), while at room temperature (25 °C), it took several days (8 days) to release the equivalent amount of DOXO just by diffusion. Along with the outstanding SAR value and the heat-triggered DOXO release, TR-DOXO are an interesting candidate for novel treatment against cancer stem cells.

### MHT Effect of TR-Cubes and TR-DOXO on CR-CSC Colonies

Due to the peculiar shape of our cubic TR-Cubes and TR-DOXO that determined their outstanding SAR values and thus heat performances in MHT, the needed dose of magnetic materials to achieve a therapeutic temperature at the tumor is one order of magnitude lower than that of spherical nanoparticles employed in clinics.^18,32,33^ Moreover, we have also previously shown by *in vivo* biodistribution studies that TR-Cubes can be renally excreted within a few months.^25^

Herein, we first look at the effects caused by MHT with TR-Cubes and then the combination of MHT and chemotherapy with TR-DOXO.

**Figure 3:**
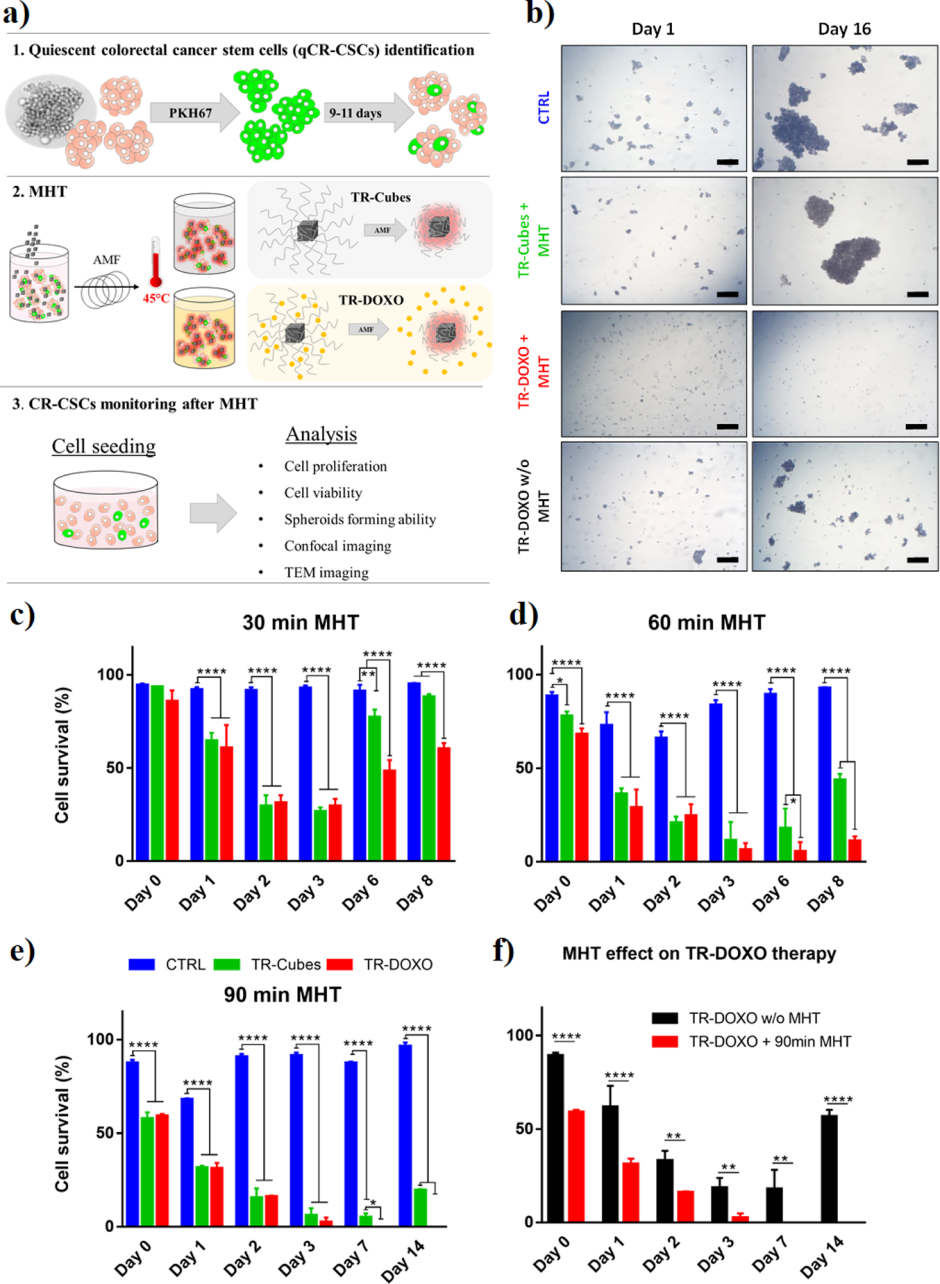
CR-CSCs #21 response to MHT alone (TR-Cubes) or combined with chemotherapy (TR-DOXO). (a) Schematic representation of the experimental protocols followed to study the effect of TR-Cubes and TR-DOXO upon exposure to MHT on CR tumor cells containing quiescent PKH^pos^ qCR-CSCs. The first step consists of identifying the qCR-CSCs followed by the use of TR-Cubes or TR-DOXO for MHT (*f* = 182 kHz with a fixed temperature of 45 °C) and finally reseeding the cells in culture to evaluate their response to the treatment *in vitro*. (b) Optical microscope representative pictures of control cells, cells exposed to MHT for 90 min (three cycles of 30 min), and cells treated with only DOXO 1 and 16 days post-treatment showing no spheroid formation for the cells treated with TR-DOXO. Scale bars: 200 μm. Viability profiles, analyzed by trypan blue assay, of the CR-CSCs treated with TR-Cubes (green bars) or TR-DOXO (red bars) after different exposure times of MHT: (c) 30 min, (d) two cycles of 30 min (2 × 30 min) for a maximum duration of 60 min, and (e) three cycles of 30 min (3 × 30 min) for a maximum duration of 90 min. The blue bars indicate the control untreated cells. (f) Viability profiles, analyzed by trypan blue assay, of the CR-CSCs treated with TR-DOXO in the presence (red bars) or absence (black bars) of MHT. Values are presented as mean with error bars indicating the standard deviation (SD) for *n* = 3 independent experiments. Statistical analysis was performed using ANOVA with a Tukey post hoc test. *p < 0.05, * *p < 0.01, ***p < 0.001, and *** *p < 0.0001.

To mimic the MHT treatment applied *in vivo*, 5 million CR-CSCs, simulating a tumor mass with a size of a few millimeters, were treated with TR-Cubes (50 μL at 4 g Fe/L or 0.2 mg Fe) or TR-DOXO (50 μL at 4 g Fe/L corresponding to 0.2 mg Fe and containing approximately 40 μg of DOXO/mg Fe) and exposed to MHT. First, we evaluated the effect of the MHT by varying the exposure time to the radiofrequency while ensuring that the therapeutic temperature reached during the MHT treatment never exceeded 45 °C. To achieve this constant temperature, the radiofrequency (*f*) was set at a clinically accepted 182 kHz and the field intensity was varied such that it never exceeded 27 kA/m (this also guaranteed that the *Hf* factor was below 5 × 10^9^ Am^–1^ s^–1^) (see Figure 3 and FigureS3). MHT cycles were applied for 10, 30, 60 (2 × 30 min), and 90 (3 × 30 min) min. The three cycles of 30 min each of MHT correspond to MHT cycles as applied in our preclinical studies.^25^ The overall cell response was followed by monitoring the cell growth rate of the spheroids (Figure 3b and Figures S4−S6) and by estimating cell survival by trypan blue assay (Figure 3c–f). When the MHT treatment exposure was increased (from 30 to 90 min), a proportional decrease in cell survival and spheroid formation was observed (Figure 3c−e and Figures S4–S6). This was also accompanied by extensive and evident signs of cellular damage after longer MHT treatments as observed by morphological cell changes under TEM analysis of treated cells (Figures S7–S11). As also indicated by cell viability and cell growth curves (Figure 3c–f and Figure S12), the cells treated with TR-Cubes and TR-DOXO did not show a significant difference in their survival profiles at the first days after MHT treatment (up to day 3). These data suggest that in the first stage post-MHT, the cells’ sufferance is mainly due to the heat therapy.

However, between days 3 and 7, a remarkable cell viability difference occurs when comparing the cells treated with TR-DOXO or TR-Cubes exposed to MHT. For the TR-DOXO treated CR-CSC samples, a significant decrease in cell survival contrasting that of the cells treated with TR-Cubes was clearly visible, suggesting a synergistic therapeutic effect of DOXO in addition to the heat generated via MHT. Figure 3c–e shows that depending on the heat stress provided by the MHT exposure, the cellular survival response is tuned and this response is delayed if stronger stress stimuli (longer MHT treatments) are provided. For the cells treated with 10 or 30 min of MHT, this effect was observed between days 2 and 5, while for the cells exposed to 60 or 90 min of MHT, a later cellular response is observed around days 7–14 post-treatment. Noteworthily, in all cases in which sole MHT therapy was applied, we observed a critical period, post-MHT, after which the cells were able to recover from the damage and start regrowing the spheroids. Differently, for the TR-DOXO at all MHT durations, a lower cell resistance (higher cell death) was observed, with complete cell death only when applying 90 min of MHT (Figure 3e). It is worth noting that for the cells treated with TR-DOXO but not exposed to MHT, the nonspecific drug release provides a reduction of cell viability, but after a week of culture, the remaining alive cells were able to restart the spheroid growth (Figure 3f). Overall, these data suggest that the samples treated with TR-DOXO are preferred to those treated with TR-Cubes and an overall MHT duration of 90 min is the most efficient treatment to reach the best therapeutic effect and completely suppress the spheroid growth. These cytotoxicity data and MHT conditions are also in agreement with the magnetic field conditions used by us in the *in vivo* efficacy study on a xenograft tumor model of adenocarcinoma when employing the same TR-DOXO.^25^

**Figure 4:**
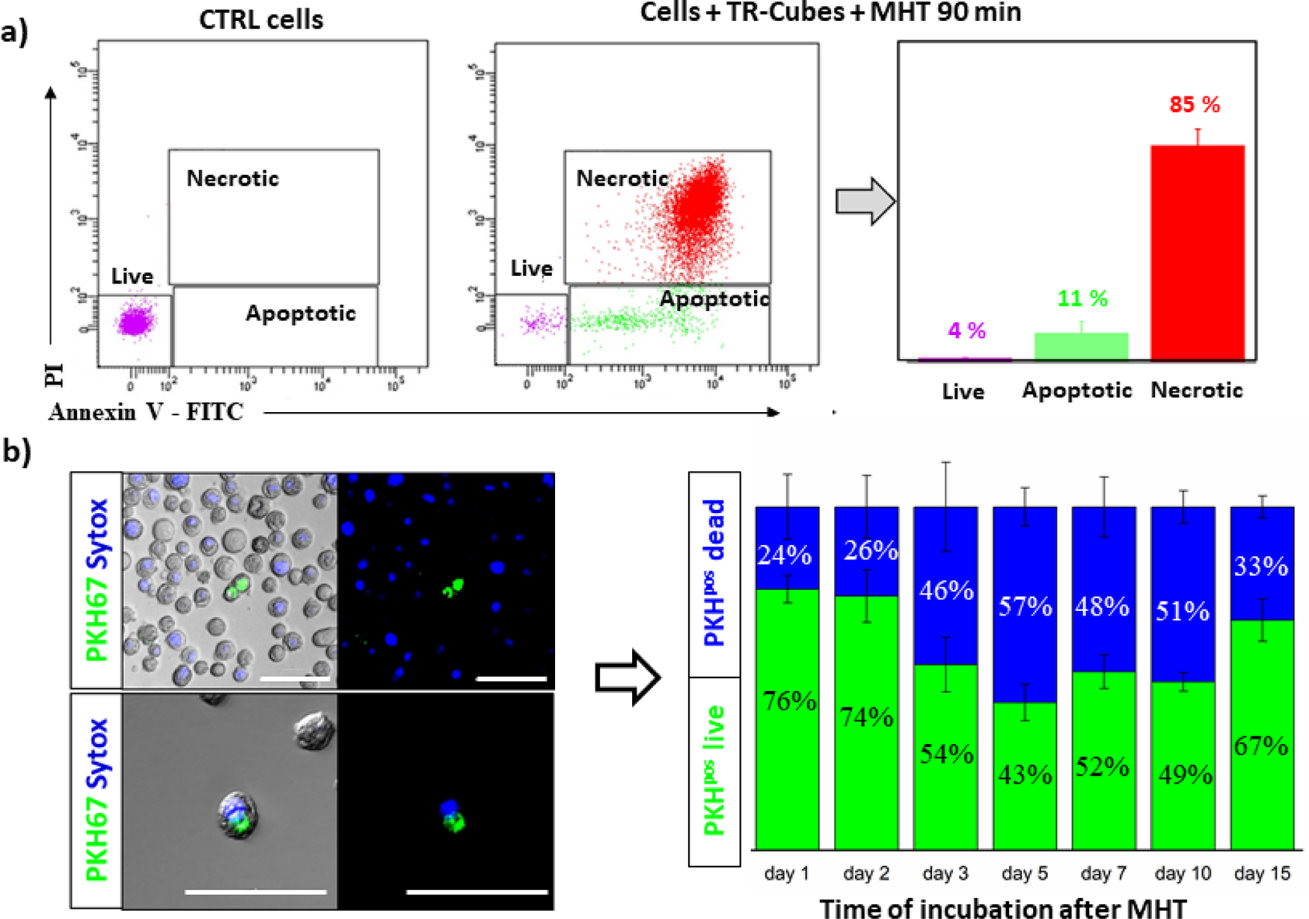
TR-Cubes MHT damage on patient CR-CSCs #21 and more specifically on qCR-CSCs #21. (a) Flow cytometry analysis of annexin V–FITC and PI staining was used to evaluate the cell viability following MHT treatment (*f* = 182 kHz reaching a fixed temperature of 45 °C for 90 min). Triplicates from the control nontreated cells and the cells exposed to MHT for 90 min were measured. The purple population in the dot plot represents live cells; green, the apoptotic fraction; and red, the dead PI-stained cells. (b) PKH^pos^ cell death estimation based on confocal microscope analysis using Sytox blue staining, specific for dead cells. Left panel, images acquired by confocal microscopy: the green signal represents PKH^pos^ cells, the blue signal represents dead cells, and colocalized green and blue represent dead PKH^pos^ cells. Right panel, cumulative representation of live (green) vs dead (blue) PKH^pos^ cell quantified from the acquired confocal images. At least 500 PKH^pos^ cells were counted per each time point in *n* = 3 independent experiments. The mean data are reported, and error bars represent SD. Scale bar of 50 μm.

To better understand the cytotoxic effects caused by the heat generated during MHT on the CR-CSCs, flow cytometry analysis was used to count the number of cells in apoptotic or necrotic stages (Figure 4a). Annexin/propidium iodide staining was conducted on the cells exposed to TR-Cubes for 90 min of MHT. Most of the CR cancer cells (>80%) died mainly by necrosis during MHT, while a small fraction was detected in the apoptotic stage (ca. 10%) and an even smaller cellular fraction was found alive (4%), which may be responsible for regrowing the spheroids (Figure 3e).

**Figure 5:**
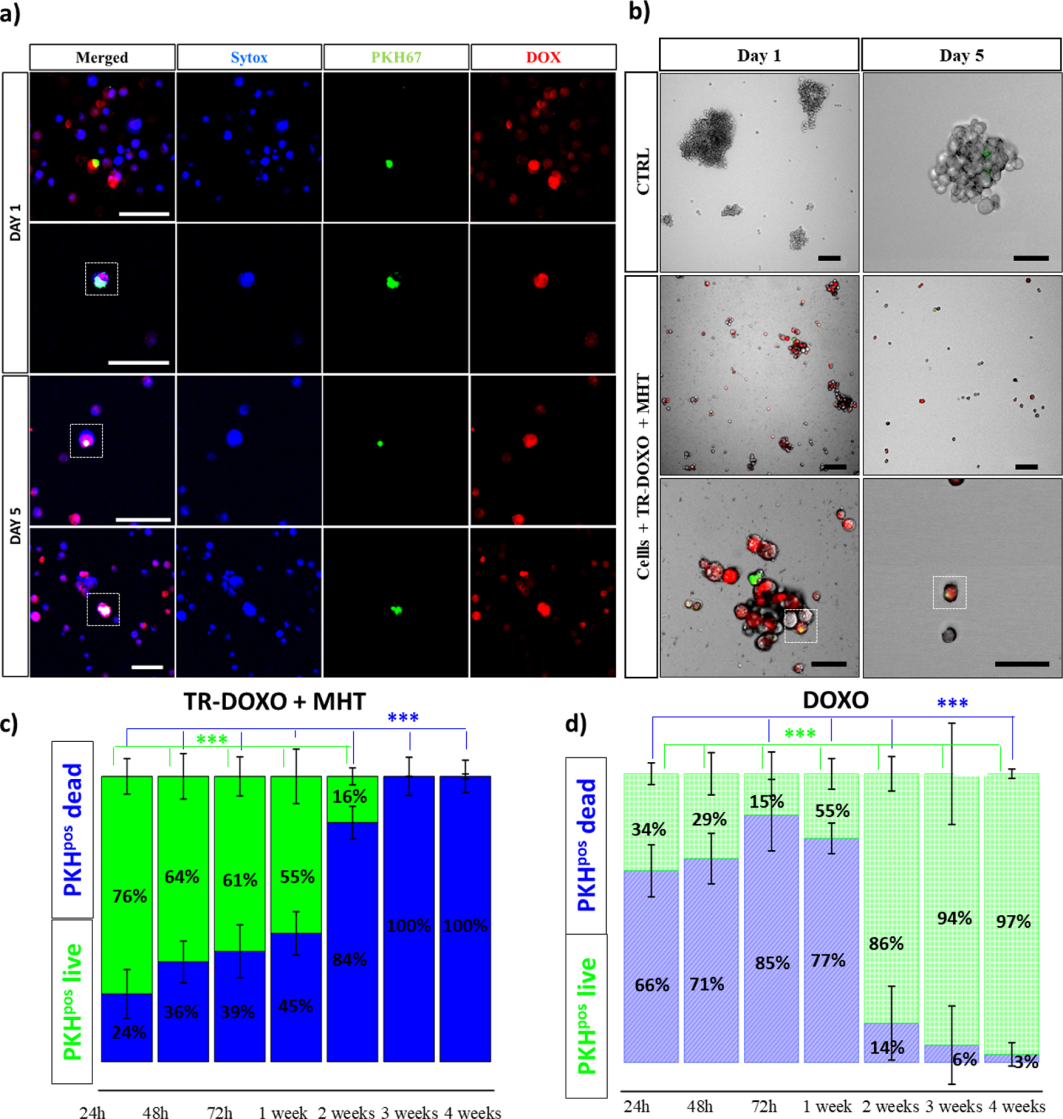
TR-DOXO dual-therapy effect on qCR-CSCs #21. (a) Confocal microscope analysis of CR-CSCs treated with TR-DOXO and exposed to MHT (90 min; three cycles of 30 min). PKH^pos^ cell (green signal), Sytox blue (blue signal) for dead cells, and uptake of DOXO (red signal). The colocalization of green, blue, and red signals at day 5 confirms the death of quiescent PKH^pos^ cells that have also internalized the released DOXO following MHT. (b) Spheroid growth monitoring after MHT (*f* = 182 kHz reaching a fixed temperature of 45 °C for 90 min; three cycles of 30 min) using TR-DOXO compared to control cells. The lack of spheroid formation on the figures on the right panel, at day 5, confirms the dual cytotoxic effects of the treatment. All scale bars are 50 μm. (c) Estimation of PKH^pos^ cell death based on confocal images acquired using Sytox blue for 4 weeks after MHT and TR-DOXO administration. (d) Estimation of PKH^pos^ cell death based on confocal images acquired using Sytox blue for 4 weeks after treatment with DOXO. For panels (c) and (d), at least 500 PKH^pos^ cells were counted per each time point and per each experimental condition in *n* = 3 independent experiments. The mean data are reported, and error bars represent SD. Statistical analysis was done using ANOVA followed by Dunn’s multiple comparison test. The statistical difference ***p < 0.001 was calculated for each time point.

To verify if, after 90 min of MHT, the quiescent PKH^pos^ green cells were a fraction of the dying population, a confocal microscopy study was conducted after co-staining the cells with Sytox blue, a marker for dead cells. The number of PKH^pos^ (green) cells colocalized with Sytox blue was counted at different time points post-MHT (Figure 4b and Figure S13). Quantitatively, out of the PKH^pos^ green-stained cells that represented the qCR-CSCs, we could count a progressively higher number of dying PKH^pos^ cells over time, reaching 50% of dead PKH^pos^ cells 5 days after MHT. This was followed by a gradual increase in the percentage of live PKH^pos^ cells between days 5 and 15 from 43 to 67% indicating that the surviving PKH^pos^ fraction started to divide and repopulate the culture. Instead, when looking at the cells exposed to the same MHT treatment using TR-DOXO, remarkable differences were observed (Figure 5). One day post-MHT treatment, a clear red signal due to DOXO internalization was already diffusively present within cells. Colocalization of DOXO (red) and PHK^pos^ cells (green) is clearly evident since each of these components occupies different subcellular compartments of the cells (DOXO in nucleus and PKH in cell membrane, Figure 5a for day 1 and also Figures S14 and S15). Additionally, after adding Sytox blue representing dead cells, at day 1, we observed lower incidences of PKH^pos^ cell death, represented by no blue co-staining with PKH^pos^ green cells. On the contrary, later by day 5, these PKH^pos^ cells showed colocalization of the blue signal for the cell death with green and red signals. This represents dead PHK^pos^ cells that had internalized DOXO after MHT (Figure5a and Figure S16).

The presence of DOXO in PKH^pos^ cells and the sign of cell sufferance that are more evident at a prolonged time post-MHT may explain why the spheroid growth is compromised when the cells are exposed to MHT using TR-DOXO (Figure 5b).

Further quantification of PKH^pos^ cells (green) and Sytox blue cells, based on the confocal images, confirmed the complete death of the qCR-CSCs after MHT with TR-DOXO (Figure 5c), contrarily to the cells treated with TR-Cubes + MHT (Figure 4b) or the cells treated only with DOXO (Figure 5d). These findings demonstrate that MHT and chemotherapy are capable of eradicating the tumor cell completely by directly affecting qCR-CSCs (PHK^pos^ cells). To test the effectiveness of our nanoplatform for a future personalized treatment, the DOXO-sensitive CR-CSC #21 used for all the above-mentioned studies was compared to another patient-derived DOXO-resistant CR-cell line, CR-CSC #27, in a long-term study conducted for at least 28 days.

**Figure 6:**
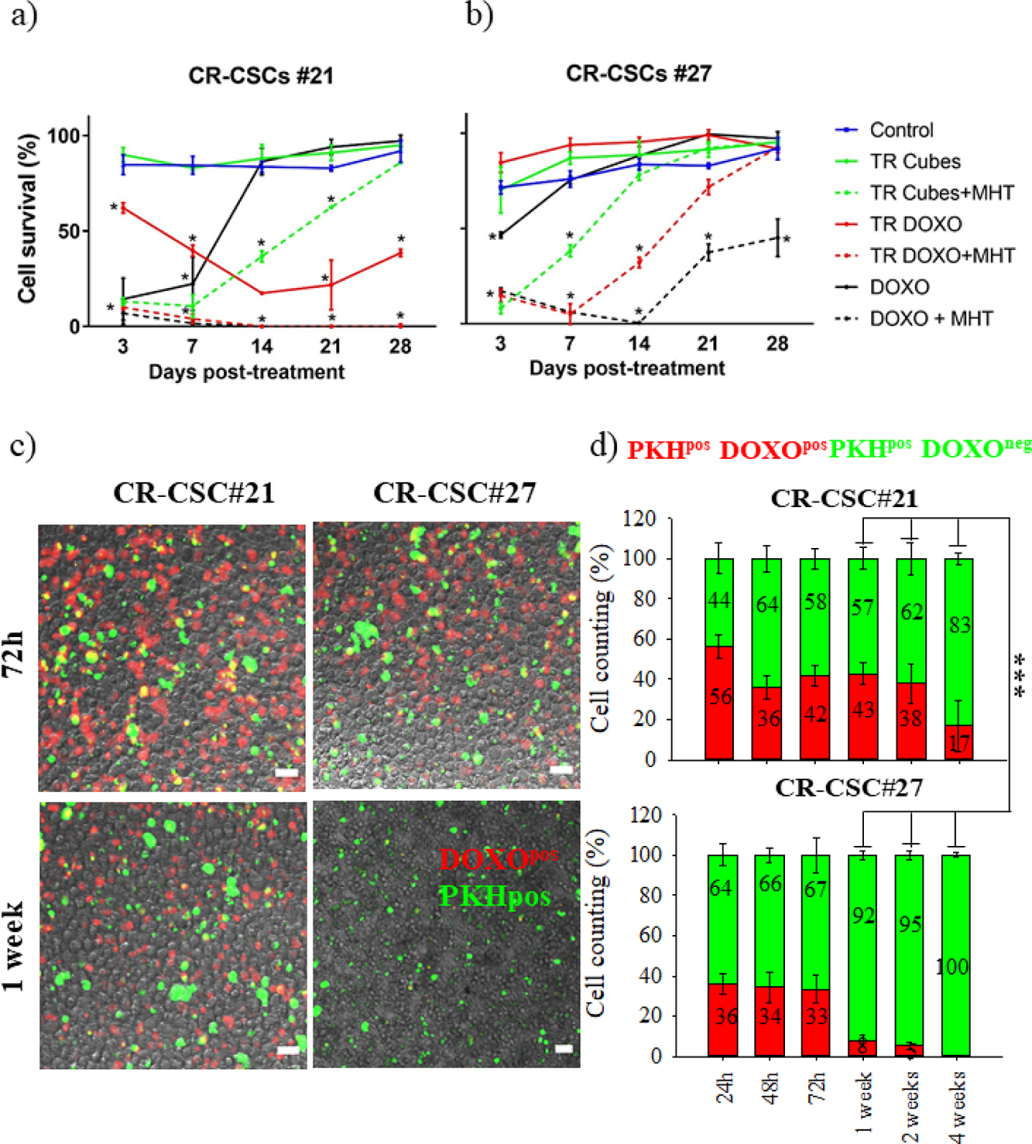
Cytotoxic effects on qCR-CSCs. Comparative cytotoxic study on DOXO-sensitive (CR-CSC #21, a) and DOXO-resistant (CR-CSC #27, b) patient cells from 24 h up to 1 month post-MHT treatment. Experimental conditions: control (not treated cells), DOXO (DOXO amount of 15 μg), TR-Cubes (cells treated only with TR-Cubes), TR-Cubes + MHT (MHT of three cycles of 30 min each at 182 kHz and 21.68 kA/m), TR-DOXO (cells treated with TR-DOXO), TR-DOXO + MHT (cells treated with TR-DOXO and exposed to MHT), and DOXO + MHT (cells treated with DOXO alone at an amount of 15 μg and exposed to MHT). Statistical analysis was done using ANOVA followed by Dunnett’s multiple comparison test. The statistical difference was calculated for each sample in each time point in comparison to the control at that same time point. The significant decrease in cell survival is annotated as * for *p* < 0.001. (c) Representative confocal pictures of CR-CSC #21 and CR-CSC #27 after 72 h and 1 week of TR-DOXO treatment showing the almost total absence of DOXO drug (red signal) inside the CR-CSC #27 line after 1 week. This is not the case for DOXO-sensitive CR-CSC #21. Green signal: PKH^pos^ cells. Scale bar: 50 μm. (d) Statistical estimation of PKHpos DOXOpos cells for CR-CSC #21 and CR-CSC #27 from 24 h until 1 month after DOXO treatment based on the images acquired. At least 500 PKH^pos^ cells were counted per each time point and per each experimental condition.***p < 0.001 when comparing qCR-CSC #21 DOXO^pos^ cells vs qCR-CSC #27 DOXO^pos^ cells at 1, 2, and 4 weeks, respectively; ANOVA test.

Besides cell viability, we have also monitored the presence of DOXO signal over time in PHK^pos^ cells. Together with TR-DOXO (see Figure 6c), free DOXO, at a dose that corresponded to the DOXO amount loaded on TR-DOXO, was also tested (it is worth noting that the amount of DOXO released by the TR-DOXO is always lower than the one loaded) (Figure S17).

As shown in Figure 6, even after 28 days, for CR-CSC #21, the complete cell growth suppression was achieved only with TR-DOXO + MHT treatment (Figure 6a). On the contrary, for DOXO-resistant CR-CSC #27, the survival rate suggests that no treatment is effective, as colonies were able to regrow after day 7 (Figure 6b). Notably, even the response to dual therapy when employing either TR-DOXO + MHT or DOXO + MHT is not enough to block the cell regrowth (Figure 6b and Figure S18).

To better understand why the dual therapy was effective for DOXO-sensitive CR-CSC #21 but not for DOXO-resistant CR-CSC #27, we focused on the effect of DOXO on the cells. On both patient cells, quantitative fluorescent analysis based on images acquired by confocal microscopy was used to estimate the number of PKH^pos^ cells (green signal) that were also DOXO positive (red signal). We found that DOXO was present from the beginning of the drug treatment on both cell types and it was still present after a month on a small fraction of CR-CSC #21-PKH^pos^ cells (ca. 17% of the cells) (Figure 6d). For CR-CSC #21, no regrowth of colony appeared up to 28 days (Figure 6a). Instead, on PKH^pos^ CR-CSC #27, where a regrowth of cell colonies appeared 7 days post-treatment (Figure 6b), by analyzing confocal images, a remarkable decrease in DOXO red signal is clearly evident between days 1 and 7 post-MHT (Figure 6c,d).

These qualitative and quantitative data confirm that for these DOXO-resistant cells, the rate of expulsion of DOXO occurs very quickly between 72 h and day 7 (less % of red signal out of the PKH^pos^ CR-CSC #27), in accordance with published data regarding the overexpression of drug-efflux transporters in CSCs.^34^ It is worth noting that even in this case to evaluate the toxicity of the treatment the main difference occurs between 3 and 7 days post-treatment. The DOXO elimination could suggest a possible mechanism of recovery of this DOXO-resistant cell line even if it went through MHT.

Next, to validate the efficiency of the dual treatment on the DOXO-resistant CR-CSC #27, we designed an experiment in which we co-administered the TR-Cubes as MHT heating agents together with oxaliplatin (OXA), a drug that can overcome drug resistance shown by CR-CSC #27 (MHT + OXA, single step). The clinically sensitive dose range of OXA for CR-CSCs as reported by Todaro *et al*. is between 5 and 100 μM.^28^

**Figure 7:**
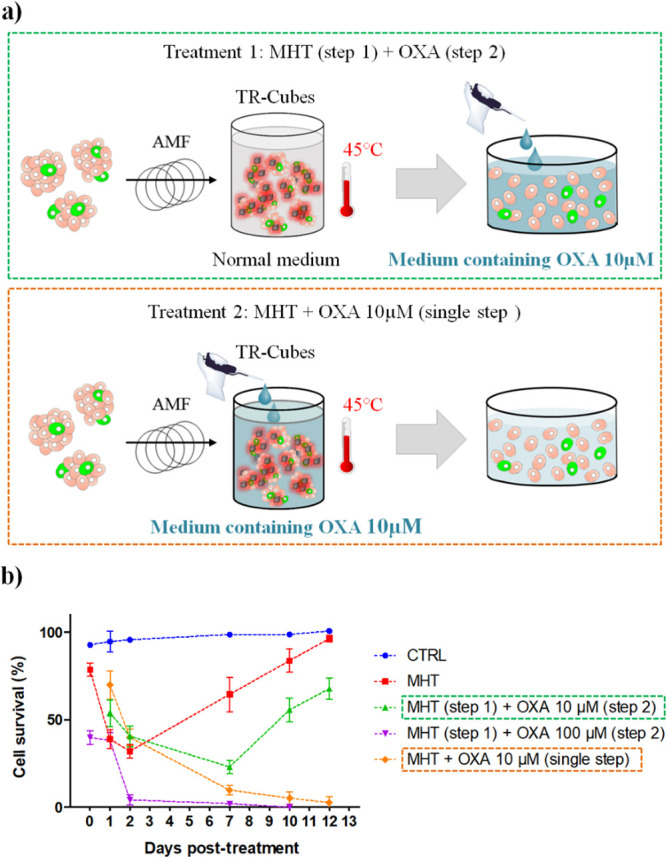
Dual-therapy effect on DOXO-resistant CR-CSCs #27 using TR-Cubes for MHT and OXA as chemotherapeutic agent. (a) Schematic diagrams of experimental treatment for MHT combined with OXA administration. (b) Viability profiles, analyzed by trypan blue assay, for the DOXO-resistant CR-CSC #27 treated with MHT using TR-Cubes (red dashed line), TR-Cubes followed by OXA administration in the medium (100 μM, purple dashed line), TR-Cubes plus OXA administration in the medium (10 μM, green dashed line), and TR-Cubes plus OXA 10 μM added during hyperthermia (orange dashed line). All MHT treatments were performed for three cycles of 30 min at 45 °C (*f* = 182 kHz, *H* ∼ 13). The blue dashed line indicates the control cells incubated at 37 °C. All values were calculated and normalized to the viability of the control cells at day 0.

We also aimed by combining the therapeutic effect of OXA and MHT to reduce the OXA dose required to achieve the elimination of CR-CSC #27. In a first set of experiments, CR-CSC #27 was first treated with TR-Cubes and exposed to MHT and then seeded in a fresh medium containing OXA at 10 or 100 μM (Figure 7a, treatment 1). Cells treated with MHT and 100 μM OXA were completely eradicated by day 7 post-treatment (Figure 7b, purple line). Contrarily, if a 10 μM OXA dose was administered to the cell media, a 70% cell mortality was reached by day 7 after treatment, after which the survival of the cells started to increase again (Figure 7b, green dashed line). Please note that cell viability was also tested for the free OXA drug in the solution at 50 μM (Figure S19). The viability data obtained are in accordance with those reported by Todaro *et al.*: there is about 60% cell death after 48 h.^28^ However, while the cells treated with 100 μM OXA were not able to recover from the treatment, the ones treated with 50 μM OXA started recovering very quickly as shown by the increase in cell viability after only 4 days post-treatment (Figure S19).

In a second set of experiments, TR-Cubes and OXA were co-administered directly to the cells before performing MHT (Figure 7a, treatment 2). Interestingly, the presence of OXA during the MHT treatment (Figure 7a, orange dashed line) brought to the complete cell elimination at a much lower OXA dose (10 μM). In fact, after 7days, the cells do not show any sign of recovery, with a complete elimination achieved by day 12 post-treatment (Figure 7b). This result indicates the importance of having the drug during the MHT as, in this case, the heat facilitates its cell internalization and enables a more effective therapeutic effect at reduced drug dosage. Our TR-DOXO nanoplatforms are indeed able to encapsulate and release the drug only upon a heat stimulus, thus enabling a significant reduction in the drug dose.

### *In Vivo* Tumorigenicity Study

**Figure 8:**
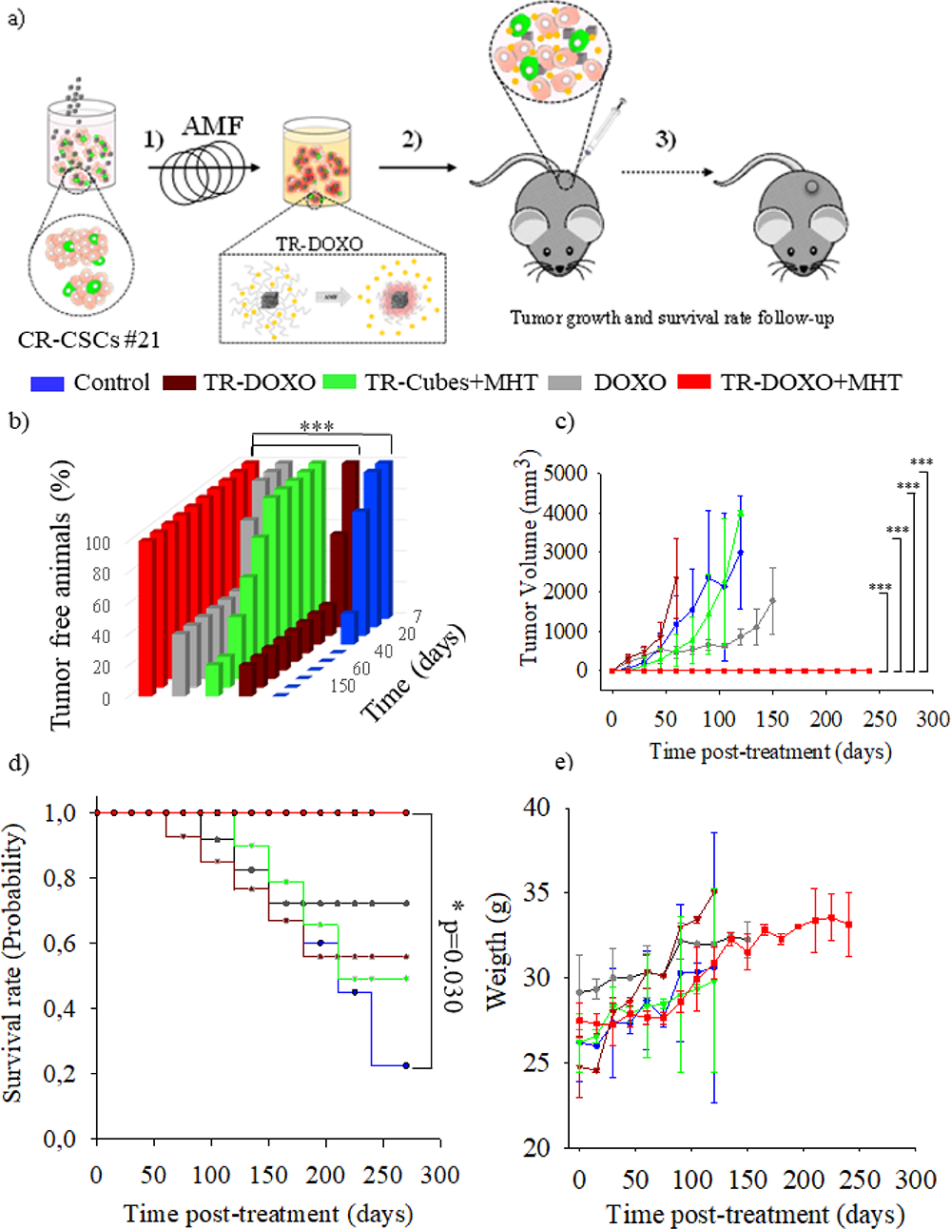
*In vivo* monitoring of tumor initiation and relapse capacity of CR-CSCs #21 after *in vitro* pretreatment with MHT using TR-Cubes or TR-DOXO nanoplatforms. (a) Scheme of the *in vivo* experiment steps. CR-CSC #21 cells subjected to administration of TR-DOXO or TR-Cubes with or without the MHT exposure (182 kHz for three cycles of MHT of 30 min each) were injected subcutaneously on the flank of NMRI nude mice (1 × 10^6^ live cells per animal). A follow-up of the animals was prolonged up to 8 months. A tumor of 2 cm in at least one of its side marked the end point of the experiment.(b) Percentage of tumor-free animals over time (8 months of experiment). * **p < 0.001 control vs TR-DOXO + MHT and ***p < 0.001 TR-DOXO vs TR-DOXO + MHT for days 120, 155, and 180, respectively; ANOVA and Tukey post hoc test. (c) Tumor growth curves (tumor volume: mm^3^) for animals injected with CR-CSC #21 cells (control, not treated) or cells previously treated with TR-Cubes + MHT, TR-DOXO, or free DOXO or TR-DOXO + MHT cells. ANOVA and Dunn post hoc test. Statistical analysis shows statistical differences, ***p < 0.001, on the experimental groups: control vs TR-DOXO + MHT, TR-Cubes + MHT vs TR-DOXO + MHT, DOXO vs TR-DOXO + MHT, and TR-DOXO vs TR- DOXO + MHT for days 45, 60, 75, 90, 105, and 120, respectively. (d) Kaplan–Meier survival plot showing the difference in tumor suppression and improved survival between the TR-DOXO + MHT and the other groups studied up to 8 months; *p = 0.030; *p* value is calculated with the log-rank test. (e) Weight graphs of animals in control (no treated), DOXO, TR-DOXO, TR-Cubes + MHT, and TR-DOXO + MHT groups showing no weight loss for the 8 months of experiment. For graphs in panels (b), (c), (d), and (e), data shown are mean ± SD, with *n* = 6 of two independent experiments.

To confirm the effective combinatorial therapeutic effects of our TR-DOXO nanoplatforms, an *in vivo* tumor formation and relapse evaluation study was performed after the cells were exposed to the treatments *in vitro*. Nanoplatforms were added to CR-CSC #21 (50 μL at 4 g Fe/L of TR-Cubes and TR-DOXO containing 50 mg of DOXO per gram of Fe) and exposed to MHT treatment at 45 °C in an *ex vivo* setup (three cycles at 182 kHz and the field intensity never exceeded 21.87 kA/m to maintain the 45 °C during the 30 min cycle). These treated cells were then injected subcutaneously in the flank of animals. With this study, the ability of CR-CSC #21 to reinitiate tumor growth was evaluated in a xenograft murine model (Figure 8a), after the cells were treated in vitro with the TR-Cubes or TR-DOXO with or without the exposure to MHT (three cycles of 30 min, at 45 °C).^19,35^ For the animal groups where no MHT exposure was foreseen, the nanoparticles were added to the cells and incubated at 37 °C for the equivalent time of MHT before proceeding with the subcutaneous injection in the right flank of the 8-week-old female animals. We then monitored the tumor formation ability, tumor volume growth, animal survival, and general animal health state. As expected only for the animal group injected with CR-CSC #21 treated with TR-DOXO and exposed to MHT (TR- DOXO + MHT), *in vivo* tumor formation results showed a complete inhibition of tumor growth for the whole duration of the experiment (250 days). Indeed, none of the animals in the group exposed to the dual therapy developed tumors throughout the whole experimental duration. This was not the case for control animals injected with nontreated CR-CSC #21, the CR-CSC #21 treated with nonspecific DOXO release (TR-DOXO with no MHT exposure), or free DOXO (the latter administered at the same dose amount that is loaded within the TR-Cubes) or for the MHT heat damage alone (TR-Cubes + MHT). Animals belonging to these groups gradually developed tumors along the experiment, with different rates of tumor growth (Figure 8b and Figure S20). Instead, only in the group of the combined treatment of TR-DOXO + MHT was the complete tumor growth suppression induced, with no detectable tumor appearing in any of the animals of this group (Figure 8c). It is worth noting that, once more, the main viability difference between treatments occurs between days 3−7 post-treatment. Surprisingly, after 150 days of cell injection, two animals in the TR-Cubes + MHT group with absolutely no visible external tumor mass demonstrated physical signs of swallowed abdomen, and thus, they were sacrificed, respecting the humane end point of the study. Post-mortem analysis of these two animals showed the presence of bloody ascites in the peritoneal cavity, and few tumor nodules were found in the mesenterium and retroperitoneum, indicating the presence of metastasis (Figure S21e).^36^ These animals however had no evidence of cachexia and weight loss. These results are indicative of the inefficiency of hyperthermia alone (TR-Cubes + MHT) to fully eradicate the qCR-CSCs: the heat can indeed delay the tumor growth, but it cannot avoid resistant tumor cells to restart growing and cause cancer relapse or even more dangerous metastasis (Figure S21e). The presence of a low percentage of animals without tumor in DOXO, TR-DOXO, and TR-Cubes + MHT groups may be due to the random presence of quiescent CSCs in the cell population initially injected in the animal.^37^

Recent reports have suggested that the percentage of CSCs within a tumor cell population of patient cells can vary from 0.02 to 25% depending on the tumor type. However, this CSC fraction resisting treatments is sufficient to restart the tumor growth by an asymmetric cell division generating the growth of self-renewing cells (carrying stemness) and general progenitor tumor cells that can further differentiate.^38−40^ Survival rate was also monitored, and mice were sacrificed when the animal reached the humanr endpoint (the tumor reached 2 cm in one of its side). Control and TR-DOXO animal groups presented a higher probability of mortality due to the fast tumor growth before 100 days post-treatment. For DOXO and TR-Cubes + MHT groups, the probability of mortality was delayed to 150 days compared to control and TR-DOXO groups. This might be explained by the slower nonspecific DOXO release from the TR-DOXO compared to administering free DOXO directly. In contrast, the CR-CSC #21 treated with TR-DOXO + MHT led to significantly enhanced overall survival (probability = 1) relative to the control groups (*p* < 0.05, *p* = 0.030). Eight months after study initiation, all the animals of this group were still alive without tumor recurrence. (Figure 8d, Figure S20). All the animals in the study groups showed an increase in body weight over time. No significant differences in the weight of the animals among different treatment groups were observed (Figure 8e).

## Conclusions

Our study demonstrates that patient-derived CR-CSCs are susceptible to the heat produced by TR-Cubes under multiple MHT exposures (3 × 30 min), at a therapeutic temperature of 45 °C, showing necrosis as the major cause of death. However, even after MHT, a small fraction of surviving qCR-CSCs can regrow the whole tumor mass. Interestingly, the longer the heat exposure time was, the longer was the delay of qCR-CSCs to reawake and restart the tumor growth (Figures 3 and 4). To inhibit the self-renewal capacity of this subcellular population, a dual therapeutic approach was required. Here, TR-DOXO, which combine the heat generated by the magnetic nanocubes under MHT with the triggered heat-mediated DOXO release, were proven to successfully eradicate qCR-CSCs (PKH^pos^ stained cells) and avoid tumor relapse (Figures 3 and 6). Under MHT, the heat produced by TR-DOXO generates a stress on the qCR-CSCs cells, inducing them to divide. At the same time, the heat facilitates DOXO diffusion and prompts drug internalization by qCR-CSCs cells, thus making them more sensitive to DOXO (Figures 5 and 6). These *in vitro* findings were also supported by tumor initiation and relapse *in vivo* studies when using patient-derived DOXO-sensitive cells (CR-CSC #21). Notably, tumor growth was completely absent only in the animals injected with the cells pretreated with TR-DOXO and exposed to MHT (TR-DOXO + MHT, Figure 8). The heat effect of TR-Cubes under MHT (TR-Cubes + MHT) was instead not able, by itself, to completely stop the tumor formation. Remarkably, in this study, the complete tumor inhibition with TR-DOXO was observed when applying MHT parameters that are used in clinics.

Importantly, we have also proved that the dual therapy can be adjusted to provide personalized therapy by choosing the most effective chemotherapeutic drug to be combined with TR-Cubes for MHT depending on the drug resistance of patient tumor cells. Indeed, for DOXO-resistant patient-derived CR-CSCs #27, the combination of MHT and DOXO did not work but the combination of MHT using TR-Cubes with OXA, another chemotherapeutic agents, could stop *in vitro* tumor cell growth (Figure 7). Interestingly, this could be achieved using a much lower dose of OXA (10 μM) than the standard OXA treatment (100 μM) only when the drug and MHT were co-administered. This outcome highlights the importance of using a smart multifunctional nanoplatform, like our TR-DOXO, to provide efficient hyperthermia and trigger chemotherapy in a single step to eliminate qCSCs.

Finally, our data suggest that it is reasonable to predict *in vitro* the cellular response of patient cells to MHT in combination with chemotherapy in a short time, with a critical time window set around 5–7 days. We consider the results obtained in this work relevant for the clinical translation of MHT therapeutic effects in combination with drug release to develop a patient-specific personalized therapy. In perspective, efforts should be made to encapsulate different chemotherapeutic drugs in such types of magnetic nanoplatform, thus providing more efficient and patient-specific therapeutic tools.

## Materials and Methods

### *In Vitro* Cell Culture

Colorectal (CR) cancer specimens were supplied by the “P. Giaccone” University Hospital following the ethical guidelines for human experimentation of the Institutional Ethics Committee. The isolation and propagation of CR-CSCs #21 and CR-CSCs #27 were performed as previously described.^29^ CR-CSCs were checked routinely for mycoplasma contamination by using the MycoAlert Plus Mycoplasma Detection Kit (Lonza). The authentication of CR-CSCs was assessed by performing a short tandem repeat (STR) analysis (GlobalFiler STR Kit, Applied Biosystems) followed by ABIPRISM 3130 genetic analyzer (Applied Biosystems) sequencing. CR-CSCs (both CR-CSC#21 and CR-CSC #27) were cultured as spheroids in Corning ultra-low attachment flasks at 37 °C and 5% CO_2_. The cells were plated at an optimal density of 1 × 10^5^ cells/mL in an Advanced DMEM/F12 medium (Gibco) freshly supplemented with HEPES (10 mM), l-glutamine (2 mM), penicillin–streptomycin (100 U/mL), *N*-acetylcysteine (1 mM), N-2 Supplement (1×), bFGF (100 ng/mL), EGF (50 ng/mL), B-27 (1×), Gastrin I (human) (10 mM), and nicotinamide (10 mM).

### Identification of Quiescent CR-CSCs: Cellular Membrane PKH Labeling

Typically, the staining was performed after the cells were in culture for at least a week. The cells were labeled with the lipophilic fluorescent dye PKH67 (Sigma) according to the manufacturer’s protocol. Briefly, cells were washed and incubated with the dye for 4 min. The staining was blocked with fetal bovine serum (FBS), and the cells were seeded in the supplemented medium as described above. The medium was changed every 2 days, the cells were examined daily by an optical microscope (Motic AE31) to confirm the spheroid formation, and pictures were acquired by the confocal microscope (A1R Resonant Confocal System on TiE inverted microscope, NIS Elements software) to follow the fluorescent signal dilution.

### Flow Cytometry of CD44v6 in PKH67-Stained CR-CSC (#21) and Stemness-Related Gene Expression Analysis

After being stained with PKH67, PKH67-stained CR-CSC (#21) cells were washed in PBS and stained with the CD44v6/APC antibody (2F10, R&D systems) or isotype matched control (IMC) (IC002A, R&D systems). Samples were analyzed using the BD FACS Melody cell sorter.

Total RNA extraction was performed by using the Trizol Reagent (Thermo Fisher). RNA samples were retrotranscribed with the iSCRIPT reverse transcription supermix kit (Bio-Rad). Real-time PCR for genes related to stemness was performed using a PrimePCR custom array (Bio-Rad). Data were analyzed with the Bio-Rad PRIME PCR analysis software.

### Fluorescent-Activated Cell Sorting (FACS) of PKH-Positive (PKH^pos^) and PKH-Negative (PKH^neg^) Cells

For PKH^pos^ and PKH^neg^ discrimination, FACS sorting was performed on a single-cell suspension 11 days after labeling with PKH using a BD FACSAriaII to obtain PKH^pos^ and PKH^neg^ cells. After gating to eliminate debris and dead cells, thresholds were set based on the signal of a control unlabeled population. All cells that were found above the established threshold were considered PKH^pos^.

### Spheroid Forming Ability of PKH-Sorted CR-CSCs

The bright fluorescent cells, based on PKH intensity (7–10%), representing the quiescent PKH^pos^ were sorted and plated as a single cell per well in a 96-well plate with an ultra-low attachment surface. Spheroid formation was observed by using the optical microscope for acquiring pictures with the Motic Images Plus 2.0 digital software and the confocal microscope.

### MHT *In Vitro* Experiments

A total of 5 × 10^6^ cells were suspended in 50 μL of the complete medium containing 4 g Fe L^–1^ of TR-Cubes or TR-DOXO. The cell suspension was exposed to an alternated magnetic field (AMF) with a fixed frequency at 182 kHz generated by a NanoScale DM100 Series (Biomagnetics Corp.). The temperature was kept at 45 °C (see Figure S3). Following the treatment, the cells were seeded in ultra-low attachment flasks at the optimal density of 6 × 10^4^ cells per mL and the viability plus spheroid growth was analyzed. Every 2 days, half of the medium was replaced with a fresh one and the cell density was diluted to half.

### Spheroid Growth Assay

After MHT, the spheroids were monitored by taking pictures using the optical microscope and by counting the total number of cells using an automated cell counter (Nucleocounter NC-100, ChemoMetec).

### Cell Viability

All methods were applied following the manufacturers’ procedures. The resistance of the CR-CSC population after exposure to MHT was evaluated by trypan blue and Sytox blue analysis. Cells were analyzed immediately after treatment and then followed for at least a week. Images were acquired using a confocal microscope (A1R Resonant Confocal System on a TiE inverted microscope; Nikon, Amsterdam, the Netherlands) for Sytox blue staining specific for dead cells (S34857, Thermo Fisher). To evaluate the induction of apoptosis/necrosis by flow cytometry dead cell apoptosis kit with annexin V–FITC and PI from Invitrogen assay was performed on triplicate samples.

### TEM Analysis of Cellular Integrity and Nanoparticle Internalization after MHT

After treatment, the cell suspension was fixed and analyzed for transmission electron microscopy (TEM). At least 500,000 cells were incubated in a growth medium supplemented with glutaraldehyde (2%) for 45 min at room temperature (RT). Cells were then centrifuged at 14,000 rpm for 10 min. The obtained pellet was then dispersed in a Na-cacodylate buffer (0.1 M, pH 7.4) supplemented with glutaraldehyde (2%) and mixed for 1 h at RT. Afterward, cells were centrifuged at 14,000 rpm for 10 min. Three washes of 10 min each were repeated in the Na-cacodylate buffer (0.1 M). Subsequently, the pellet was incubated with the Na-cacodylate buffer (0.1 M) supplemented with OsO_4_ (1%) for 1 h at RT. Three washes of 10 min each in the Na-cacodylate buffer (0.1 M) were performed. Then, the pellet was washed three times for 5 min with mQ water and incubated overnight (ON) in a uranyl acetate buffer 1% (in water). Next, the sample was gradually dehydrated in ethanol (EtOH) at increasing concentrations of 70, 90, 96, and 100%. Then, the pellet was washed three times for 15 min with propylene oxide. Afterward, samples were incubated in a solution of Spurr and propylene oxide (1:3) and for 3 h in a Spurr and propylene oxide solution (1:1). Finally, the pellet was incubated for 3 h in Spurr and included into it by curing at 70 °C ON. A thin section of 70 nm of selected zones was observed with the JEOL Jem1011 electron microscope operated at 100 keV.

### *In Vivo* Studies

The study was carried out in compliance with the protocol approved by the Italian Ministry of Health Protocol No. 059 according to the DLgs 116/1992. Pathogen-free 8-week-old female immunodeficient athymic NMRI nude mice were used for all the procedures. They were housed in IVC cages in a temperature-controlled room with a 12/12-h dark/light cycle, with *ad libitum* access to water and food. A total of 1 × 10^6^ CR-CSC #21 colorectal cancer patient cells, which were previously treated with TR-DOXO (50 μL at 4 g Fe/L that corresponds to 0.2 mg Fe and containing approximately 8 μg of DOXO) and exposed to an alternating magnetic field of 22 kA/m and to a frequency of 182 kHz for 90 min (three cycles of 30 min each) (TR-DOXO + MHT experimental group), were injected subcutaneously in 150 μL of the complete cell culture medium (DMEM-F12 and Matrigel (1:1)) in the flank of each animal to induce the xenograft model. Animals that received a similar number of untreated cells (Control) or cells treated previously with TR-DOXO cubes only (50 μL at 4 g Fe/L that corresponds to 0.2 mg Fe and containing approximately 8 μg of DOXO), the TR-DOXO experimental group, DOXO free-drug (10 μg DOXO) (DOXO experimental group), or previously treated with of TR-Cubes (50 μL at 4 g Fe/L that corresponds to 0.2 mg Fe) and exposed for 90 min to an alternating magnetic field (TR-Cubes + MHT experimental group), served as controls (*n* = 6 mice/group). Animals were observed every 3 days for the appearance of palpable tumors. Tumor dimensions were measured using a digital caliper once a week, and they were calculated using the following formula: *V* = (*a* × *b*^2^)/2, where *a* = length and *b* = width. The following marked the end of the study for each animal: when the tumor size reached 1.5–2 cm/side or when the tumors became ulcerated. Weight values of the animals were also recorded at the same time as the tumoral volume measurement. It was considered by veterinary advice that all the animals with a decrease of 20% in weight should be sacrificed in compliance with animal welfare rules.

### Survival Study

Kaplan–Meier survival analysis was performed to plot the time vs the fraction of all individuals surviving as of that time. Each step in the curve marks an event in the study, which, in this case, is when the animal was sacrificed as a result of the tumor lengths reaching 2 cm/side. This curve was generated in the SigmaPlot software with a 95% confidence interval for fractional survival at any particular time.

### Statistics

For *in vitro* studies, *n* = 3 and the statistical calculations within multiple groups were done using ANOVA and Tukey’s, or ANOVA and Dunnett’s multiple comparison test, as specified in the caption of each figure, with 95% confidence interval using the GraphPad or SigmaPlot software. Significant differences were reported as ***p < 0.001 unless reported otherwise in the figures.

For *in vivo* experiments, *n* = 6 animals per experimental condition were used. Randomization was used to allocate animals in different groups for the nude mice xenograft of CR-CSC #21 using the EXCEL software method. For the tumor growth and weight curve, the mean and SD of the tumor volume/animal weights at the end point of each group were analyzed using two-way ANOVA and pairwise multiple comparison procedures (Dunn’s method) post hoc nonparametric test. The survival analysis of the animals was done using a Kaplan–Meier survival plot in the SigmaPlot software. *p* value was calculated with the log-rank test.

## Supporting Information Available

The Supporting Information is available free of charge on the ACS Publications Web site at DOI: Additional figures, as described in the text, include results from colony-forming assays, CSC gene expression studies, nanocube heating profiles, TEM image analysis of the cells after MHT, cellular growth curves, confocal images, cell death studies, and *in vivo* qualitative studies (Figures S1–S21) (PDF)

## References

[ref1] Meacham C. E.; Morrison S. J. Tumour Heterogeneity and Cancer Cell Plasticity. Nature 2013, 501, 328–337. 10.1038/nature12624. 24048065 PMC4521623

[ref2] Reya T.; Morrison S. J.; Clarke M. F.; Weissman I. L. Stem Cells, Cancer, and Cancer Stem Cells. Nature 2001, 414, 105–111. 10.1038/35102167. 11689955

[ref3] Luna J. I.; Grossenbacher S. K.; Murphy W. J.; Canter R. J. Targeting Cancer Stem Cells with Natural Killer Cell Immunotherapy. Expert Opin. Biol.Ther. 2017, 17, 313–324. 10.1080/14712598.2017.1271874. 27960589 PMC5311007

[ref4] Ho P. L.; Kurtova A.; Chan K. S. Normal and Neoplastic Urothelial Stem Cells: Getting To the Root of the Problem. Nat. Rev. Urol. 2012, 9, 583–594. 10.1038/nrurol.2012.142. 22890301 PMC3468664

[ref5] Batlle E.; Clevers H. Cancer Stem Cells Revisited. Nat. Med. 2017, 23, 1124–1134. 10.1038/nm.4409. 28985214

[ref6] Di Franco S.; Todaro M.; Dieli F.; Stassi G. Colorectal Cancer Defeating? Challenge Accepted!. Mol Aspects Med 2014, 39, 61–81. 10.1016/j.mam.2013.07.001. 23927966

[ref7] Flemming A. Targeting The Root of Cancer Relapse. Nat. Rev. Drug Discov 2015, 14, 165 10.1038/nrd4560. 25722238

[ref8] Beck B.; Blanpain C. Unravelling Cancer Stem Cell Potential. Nat. Rev. Cancer 2013, 13, 727–738. 10.1038/nrc3597. 24060864

[ref9] Lombardo Y.; Scopelliti A.; Cammareri P.; Todaro M.; Iovino F.; Ricci-Vitiani L.; Gulotta G.; Dieli F.; de Maria R.; Stassi G. Bone Morphogenetic Protein 4 Induces Differentiation of Colorectal Cancer Stem Cells and Increases Their Response to Chemotherapy in Mice. Gastroenterology 2011, 140, 297–309.e6. 10.1053/j.gastro.2010.10.005. 20951698

[ref10] Oei A. L.; Vriend L. E. M.; Krawczyk P. M.; Horsman M. R.; Franken N. A. P.; Crezee J. Targeting Therapy-Resistant Cancer Stem Cells by Hyperthermia. Int. J. Hyperthermia 2017, 33, 419–427. 10.1080/02656736.2017.1279757. 28100096

[ref11] Chen W.; Dong J.; Haiech J.; Kilhoffer M.-C.; Zeniou M. Cancer Stem Cell Quiescence and Plasticity as Major Challenges in Cancer Therapy. Stem Cells Int. 2016, 2016, 1 10.1155/2016/1740936. PMC4932171 27418931

[ref12] Moroz P.; Jones S. K.; Gray B. N. Magnetically Mediated Hyperthermia: Current Status and Future Directions. Int. J. Hyperthermia 2002, 18, 267–284. 10.1080/02656730110108785. 12079583

[ref13] Issels R. D.; Lindner L. H.; Verweij J.; Wust P.; Reichardt P.; Schem B.-C.; Abdel-Rahman S.; Daugaard S.; Salat C.; Wendtner C.-M.; Vujaskovic Z.; Wessalowski R.; Jauch K.-W.; Dürr H. R.; Ploner F.; Baur-Melnyk A.; Mansmann U.; Hiddemann W.; Blay J.-Y.; Hohenberger P.; Neo-Adjuvant Chemotherapy Alone or With Regional Hyperthermia for Localised High-Risk Soft-Tissue Sarcoma: a Randomised Phase 3 Multicentre Study. Lancet Oncol. 2010, 11, 561–570. 10.1016/S1470-2045(10)70071-1. 20434400 PMC3517819

[ref14] Mai B. T.; Fernandes S.; Balakrishnan P. B.; Pellegrino T. Nanosystems Based on Magnetic Nanoparticles and Thermo- or pH-Responsive Polymers: an Update and Future Perspectives. Acc. Chem. Res. 2018, 51, 999–1013. 10.1021/acs.accounts.7b00549. 29733199

[ref15] van der Zee J. Heating the Patient: a Promising Approach?. Ann. Oncol. 2002, 13, 1173–1184. 10.1093/annonc/mdf280. 12181239

[ref16] Wust P.; Hildebrandt B.; Sreenivasa G.; Rau B.; Gellermann J.; Riess H.; Felix R.; Schlag P. M. Hyperthermia in Combined Treatment of Cancer. Lancet Oncol. 2002, 3, 487–497. 10.1016/S1470-2045(02)00818-5. 12147435

[ref17] Luo S. W. L.; Ding W.; Wang H.; Zhou J.; Jin H.; Su S.; Ouyang W. Clinical Trials of Magnetic Induction Hyperthermia for Treatment of Tumours. OA Cancer 2014, 2.

[ref18] Maier-Hauff K.; Ulrich F.; Nestler D.; Niehoff H.; Wust P.; Thiesen B.; Orawa H.; Budach V.; Jordan A. Efficacy and Safety of Intratumoral Thermotherapy Using Magnetic Iron-Oxide Nanoparticles Combined With External Beam Radiotherapy on Patients with Recurrent Glioblastoma Multiforme. J. Neuro-Oncol. 2011, 103, 317–324. 10.1007/s11060-010-0389-0. PMC3097345 20845061

[ref19] Burke A. R.; Singh R. N.; Carroll D. L.; Wood J. C. S.; D’Agostino R. B.; Ajayan P. M.; Torti F. M.; Torti S. V. The Resistance of Breast Cancer Stem Cells to Conventional Hyperthermia and Their Sensitivity to Nanoparticle-Mediated Photothermal Therapy. Biomaterials 2012, 33, 2961–2970. 10.1016/j.biomaterials.2011.12.052. 22245557 PMC3286596

[ref20] Atkinson R. L.; Zhang M.; Diagaradjane P.; Peddibhotla S.; Contreras A.; Hilsenbeck S. G.; Woodward W. A.; Krishnan S.; Chang J. C.; Rosen J. M. Thermal Enhancement with Optically Activated Gold Nanoshells Sensitizes Breast Cancer Stem Cells to Radiation Therapy. Sci. Transl. Med. 2010, 2, 55ra79 10.1126/scitranslmed.3001447. PMC4123313 20980696

[ref21] Sadhukha T.; Niu L.; Wiedmann T. S.; Panyam J. Effective Elimination of Cancer Stem Cells by Magnetic Hyperthermia. Mol. Pharmaceutics 2013, 10, 1432–1441. 10.1021/mp400015b. 23432410

[ref22] Kwon Y.-S.; Sim K.; Seo T.; Lee J.-K.; Kwon Y.; Yoon T.-J. Optimization of Magnetic Hyperthermia Effect for Breast Cancer Stem Cell Therapy. RSC Adv. 2016, 6, 107298–107304. 10.1039/C6RA22382F.

[ref23] Hergt R.; Dutz S. Magnetic Particle Hyperthermia—Biophysical Limitations of a Visionary Tumour Therapy. J. Magn. Magn. Mater. 2007, 311, 187–192. 10.1016/j.jmmm.2006.10.1156.

[ref24] Liu D.; Hong Y.; Li Y.; Hu C.; Yip T.-C.; Yu W.-K.; Zhu Y.; Fong C.-C.; Wang W.; Au S.-K.; Wang S.; Yang M. Targeted Destruction of Cancer Stem Cells Using Multifunctional Magnetic Nanoparticles That Enable Combined Hyperthermia and Chemotherapy. Theranostics 2020, 10, 1181–1196. 10.7150/thno.38989. 31938059 PMC6956796

[ref25] Mai B. T.; Balakrishnan P. B.; Barthel M. J.; Piccardi F.; Niculaes D.; Marinaro F.; Fernandes S.; Curcio A.; Kakwere H.; Autret G.; Cingolani R.; Gazeau F.; Pellegrino T. Thermoresponsive Iron Oxide Nanocubes for an Effective Clinical Translation of Magnetic Hyperthermia and Heat-Mediated Chemotherapy. ACS Appl. Mater. Interfaces 2019, 11, 5727–5739. 10.1021/acsami.8b16226. 30624889 PMC6376448

[ref26] de Oliveira Silva J.; Fernandes R. S.; Ramos Oda C. M.; Ferreira T. H.; Machado Botelho A. F.; Martins Melo M.; de Miranda M. C.; Assis Gomes D.; Dantas Cassali G.; Townsend D. M.; Rubello D.; Oliveira M. C.; de Barros A. L. B. Folate-Coated, Long-Circulating and ph-Sensitive Liposomes Enhance Doxorubicin Antitumor Effect in a Breast Cancer Animal Model. Biomed. Pharmacother. 2019, 118, 109323 10.1016/j.biopha.2019.109323. 31400669 PMC7104811

[ref27] Alberini J.-L.; Boisgard R.; Guillermet S.; Siquier K.; Jego B.; Thézé B.; Urien S.; Rezaï K.; Menet E.; Maroy R.; Dollé F.; Kühnast B.; Tavitian B. Multimodal In Vivo Imaging of Tumorigenesis and Response to Chemotherapy in a Transgenic Mouse Model of Mammary Cancer. Mol. Imaging Biol. 2016, 18, 617–626. 10.1007/s11307-015-0916-7. 26630973 PMC4927598

[ref28] Todaro M.; Alea M. P.; Di Stefano A. B.; Cammareri P.; Vermeulen L.; Iovino F.; Tripodo C.; Russo A.; Gulotta G.; Medema J. P.; Stassi G. Colon Cancer Stem Cells Dictate Tumor Growth and Resist Cell Death by Production of Interleukin-4. Cell Stem Cell 2007, 1, 389–402. 10.1016/j.stem.2007.08.001. 18371377

[ref29] Todaro M.; Gaggianesi M.; Catalano V.; Benfante A.; Iovino F.; Biffoni M.; Apuzzo T.; Sperduti I.; Volpe S.; Cocorullo G.; Gulotta G.; Dieli F.; De Maria R.; Stassi G. CD44v6 Is a Marker of Constitutive and Reprogrammed Cancer Stem Cells Driving Colon Cancer Metastasis. Cell Stem Cell 2014, 14, 342–356. 10.1016/j.stem.2014.01.009. 24607406

[ref30] Pece S.; Tosoni D.; Confalonieri S.; Mazzarol G.; Vecchi M.; Ronzoni S.; Bernard L.; Viale G.; Pelicci P. G.; Di Fiore P. P. Biological and Molecular Heterogeneity of Breast Cancers Correlates with Their Cancer Stem Cell Content. Cell 2010, 140, 62–73. 10.1016/j.cell.2009.12.007. 20074520

[ref31] Pastò A.; Marchesi M.; Diamantini A.; Frasson C.; Curtarello M.; Lago C.; Pilotto G.; Parenti A. R.; Esposito G.; Agostini M.; Nitti D.; Amadori A. PKH26 Staining Defines Distinct Subsets of Normal Human Colon Epithelial Cells at Different Maturation Stages. PLoS One 2012, 7, e43379 10.1371/journal.pone.0043379. 22927961 PMC3425557

[ref32] Kolosnjaj-Tabi J.; Di Corato R.; Lartigue L.; Marangon I.; Guardia P.; Silva A. K. A.; Luciani N.; Clément O.; Flaud P.; Singh J. V.; Decuzzi P.; Pellegrino T.; Wilhelm C.; Gazeau F. Heat-Generating Iron Oxide Nanocubes: Subtle “Destructurators” of the Tumoral Microenvironment. ACS Nano 2014, 8, 4268–4283. 10.1021/nn405356r. 24738788

[ref33] Guardia P.; Di Corato R.; Lartigue L.; Wilhelm C.; Espinosa A.; Garcia-Hernandez M.; Gazeau F.; Manna L.; Pellegrino T. Water-Soluble Iron Oxide Nanocubes with High Values of Specific Absorption Rate for Cancer Cell Hyperthermia Treatment. ACS Nano 2012, 6, 3080–3091. 10.1021/nn2048137. 22494015

[ref34] Turdo A.; Veschi V.; Gaggianesi M.; Chinnici A.; Bianca P.; Todaro M.; Stassi G. Meeting the Challenge of Targeting Cancer Stem Cells. Front. Cell Dev. Biol. 2019, 7, 16 10.3389/fcell.2019.00016. 30834247 PMC6387961

[ref35] Yang R.; An L. Y.; Miao Q. F.; Li F. M.; Han Y.; Wang H. X.; Liu D. P.; Chen R.; Tang S. Q. Effective Elimination of Liver Cancer Stem-Like Cells by CD90 Antibody Targeted Thermosensitive Magnetoliposomes. Oncotarget 2016, 7, 35894–35916. 10.18632/oncotarget.9116. 27145285 PMC5094971

[ref36] Higuchi T.; Yokobori T.; Naito T.; Kakinuma C.; Hagiwara S.; Nishiyama M.; Asao T. Investigation into Metastatic Processes and the Therapeutic Effects of Gemcitabine on Human Pancreatic Cancer Using an Orthotopic SUIT-2 Pancreatic Cancer Mouse Model. Oncol Lett 2018, 15, 3091–3099. 10.3892/ol.2017.7722. 29435042 PMC5778887

[ref37] Mummery C.; Van de Stolpe A.; Roelen B.; Clevers H., Stem Cells: Scientific Facts and Fiction. Academic Press: 2014.

[ref38] Toledo-Guzmán M. E.; Bigoni-Ordóñez G. D.; Ibáñez Hernández M.; Ortiz-Sánchez E. Cancer Stem Cell Impact on Clinical Oncology. World J Stem Cells 2018, 10, 183–195. 10.4252/wjsc.v10.i12.183. 30613312 PMC6306557

[ref39] Bu P.; Chen K.-Y.; Lipkin S. M.; Shen X. Asymmetric Division: a Marker for Cancer Stem Cells in Early Stage Tumors?. Oncotarget 2013, 4, 950–951. 10.18632/oncotarget.1029. 23807730 PMC3759670

[ref40] Meng M.; Zhao X.-H.; Ning Q.; Hou L.; Xin G.-H.; Liu L.-F. Tumor Stem Cells: a New Approach for Tumor Therapy (Review). Oncol Lett 2012, 4, 187–193. 10.3892/ol.2012.730. 22844351 PMC3402726

