# Supplementary material for: Magnetic Nanoparticle-Based Hyperthermia Mediates Drug Delivery and Impairs the Tumorigenic Capacity of Quiescent Colorectal Cancer Stem Cells
Source: ACS Appl Mater Interfaces. 2021 Apr 2;13(14):15959–72. doi: 10.1021/acsami.0c21349 (PMC8045020; doi:10.1021/acsami.0c21349)
Supplement: Supplementary file 1 — am0c21349_si_001.pdf [file am0c21349_si_001.pdf]

Supporting information

Magnetic nanoparticle-based hyperthermia mediates drug delivery and impairs the tumorigenic capacity of quiescent colorectal cancer stem cells

Soraia Fernandes,[1&#] Tamara Fernandez,[1&] Sabrina Metze,[1] Preethi B Balakrishnan,[1%] Binh T. Mai,[1] John Conteh,[1] Claudia De Mei,[1] Alice Turdo,[2] Simone Di Franco,[3] Giorgio Stassi,[3] Matilde Todaro,[2] Teresa Pellegrino[1]*

1 Istituto Italiano di Tecnologia (IIT), via Morego 30, 16163 Genova, Italy
2 PROMISE Department, Piazza delle Cliniche 2, University of Palermo, 90133, Palermo, Italy
3 DICHIRONS Department, Via del Vespro 129, University of Palermo, 90133, Palermo,Italy
[#]current affiliation: International Clinical Research Center (FNUSA-ICRC), St. Anne's University Hospital, Brno, Czech Republic
% current affiliation: George Washington Cancer Center, The George Washington University, 800 22nd ST NW, Washington DC 20052, USA
& these authors contributed equally to this work

*Correspondence and requests for materials should be addressed to TP: teresa.pellegrino@iit.it

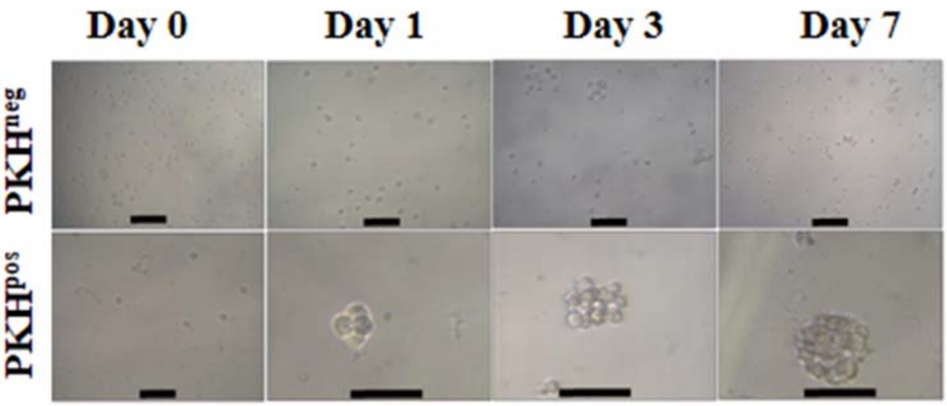

Figure S1. a) Colony forming assay comparison for PKH^{pos} and PKH^{neg} cells after FACS sorting. Scale bars: 50 µm.

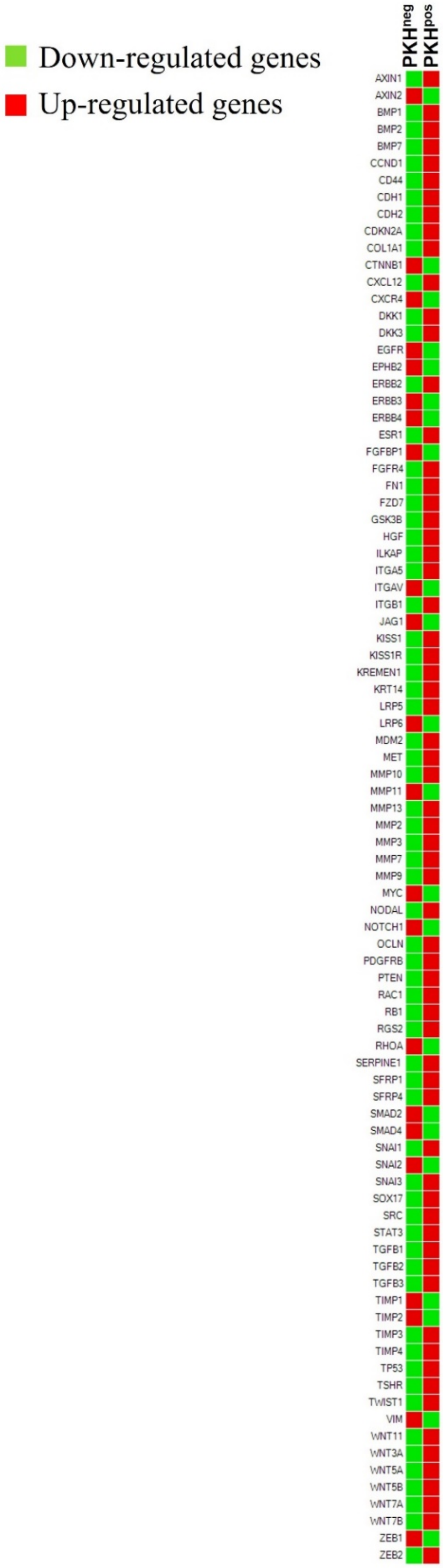

Figure S2. Clustergrams of up- and downregulated stemness-related genes in FACS-sorted PKH^{pos} and PKH^{neg} CR-CSCs following 11 days of PKH67 staining

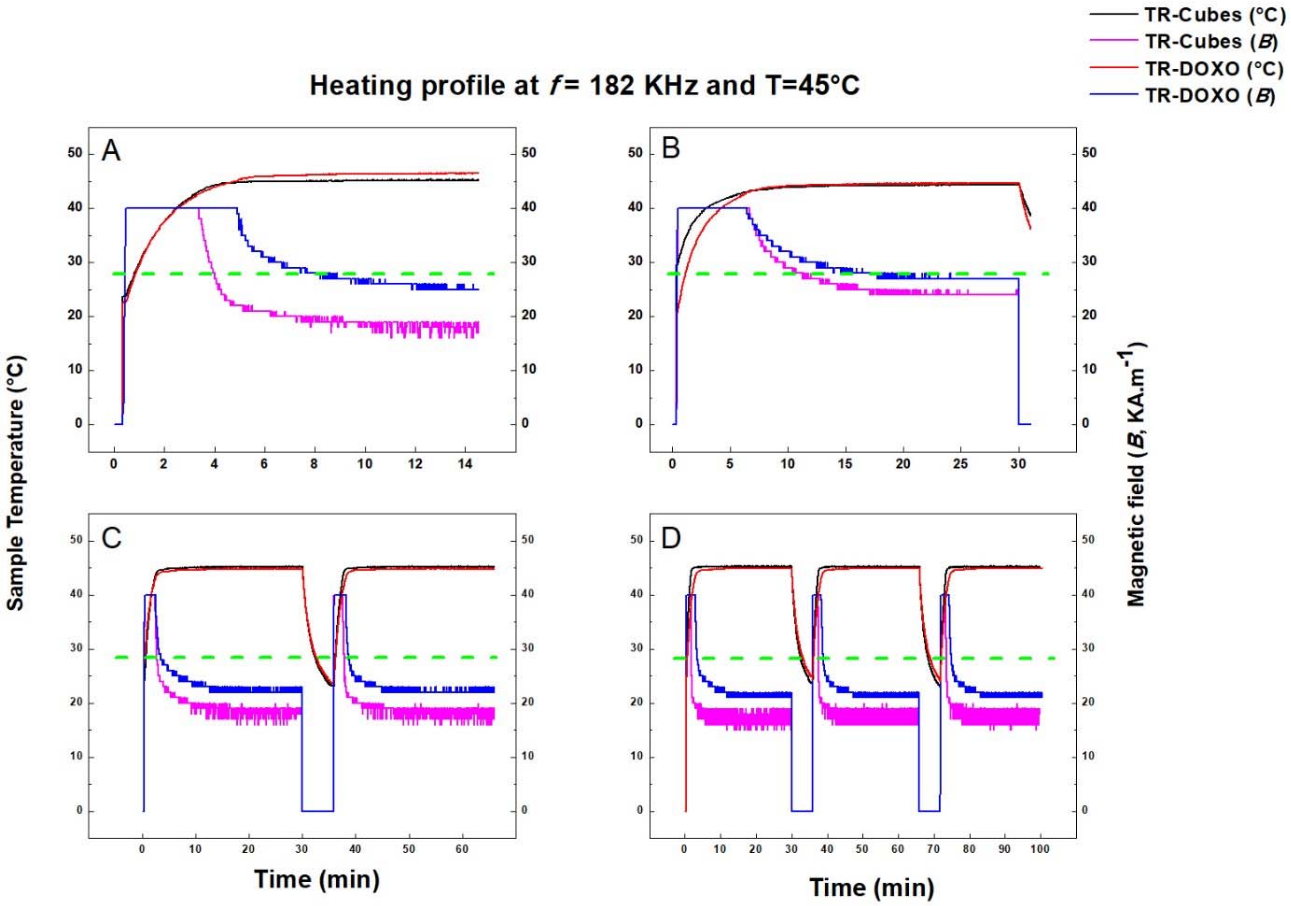

Figure S3. Heat profiles of the TR-Cubes (black line) and TR-DOXO (red line) for the different MHT applied: A) 10 minutes; B) 30 minutes; C) 60 minutes; D) 90 minutes. All treatements were performed at the fixed frequency of 182 KHz. The blue line is the profile of the field amplitude applied to the TR-Cubes and the violet one for the TR-DOXO. The green dashed line indicates the treshold for the maximum field intensity possible in order to not overcome the Hf product clinically acceptable.

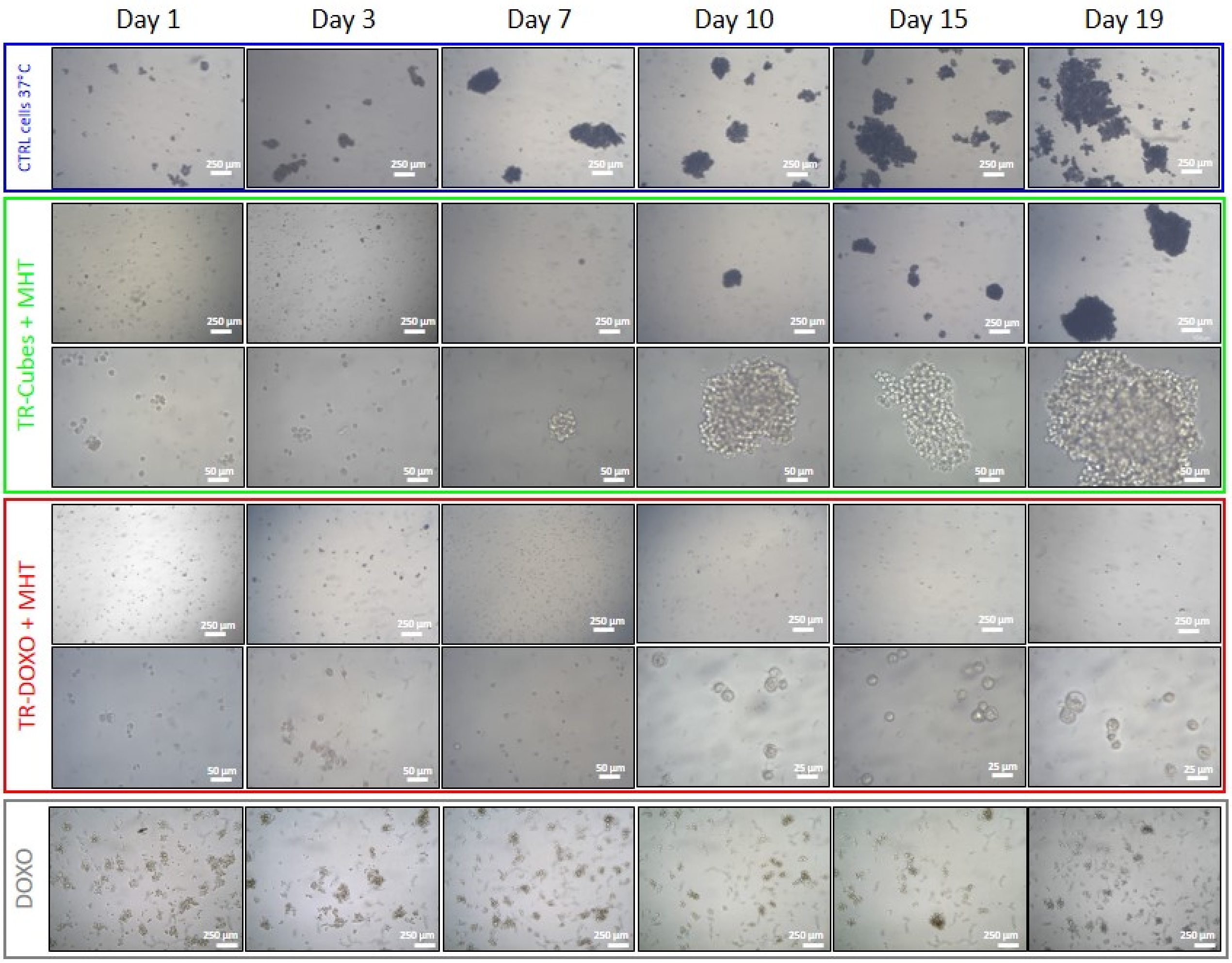

Figure S4. Cell growth and spheroids formation of CR-CSC #21 after MHT at 45°C for 90 minutes, using TR-Cubes or TR-DOXO. The last row of pictures represents the spheroids treated with DOXO, showing thtat the drug alone is not enough to completely impair colony growth. The images were acquired using an optical microscope coupled with a camera.

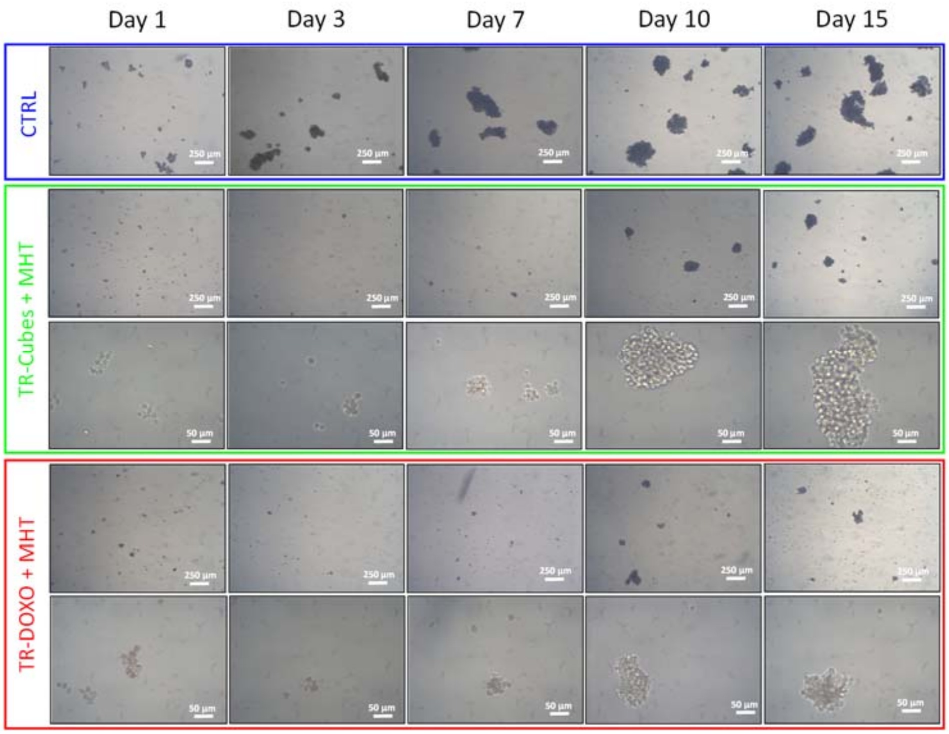

Figure S5. Cell growth and spheroids formation of CR-CSC #21 after MHT at 45°C for 60 minutes, using TR-Cubes or TR-DOXO. The images were acquired using an optical microscope coupled with a camera.

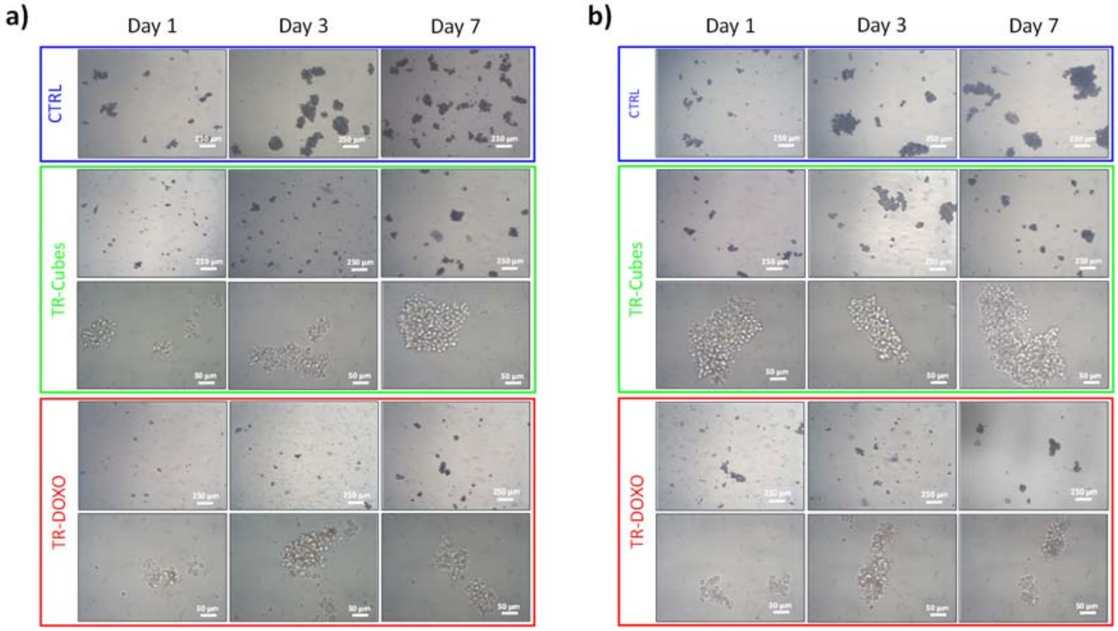

Figure S6. Cell growth and spheroids formation of CR-CSC #21 after MHT at 45°C, for a) 30 minutes and b) 10 minutes, using TR-Cubes or TR-DOXO. The images were acquired using an optical microscope coupled with a camera.

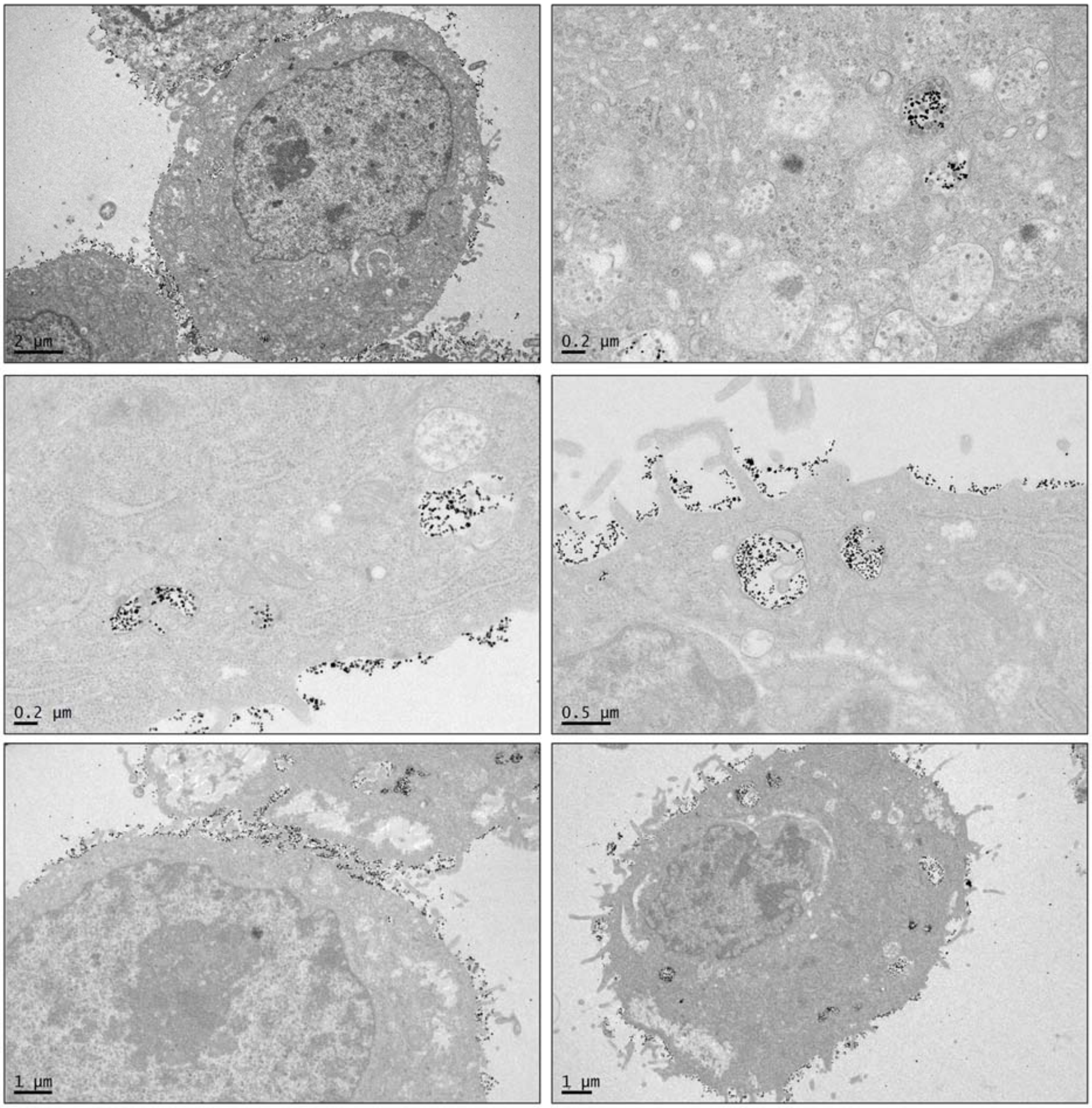

Figure S7. TEM analysis of the CR-CSC #21 cells treated with TR-DOXO at 37ºC (no MHT was applied). The cells were processed after 1 day of incubation with the TR-DOXO. The images highlight TR-DOXO internalization while minimal or negligible cellular damage is observed.

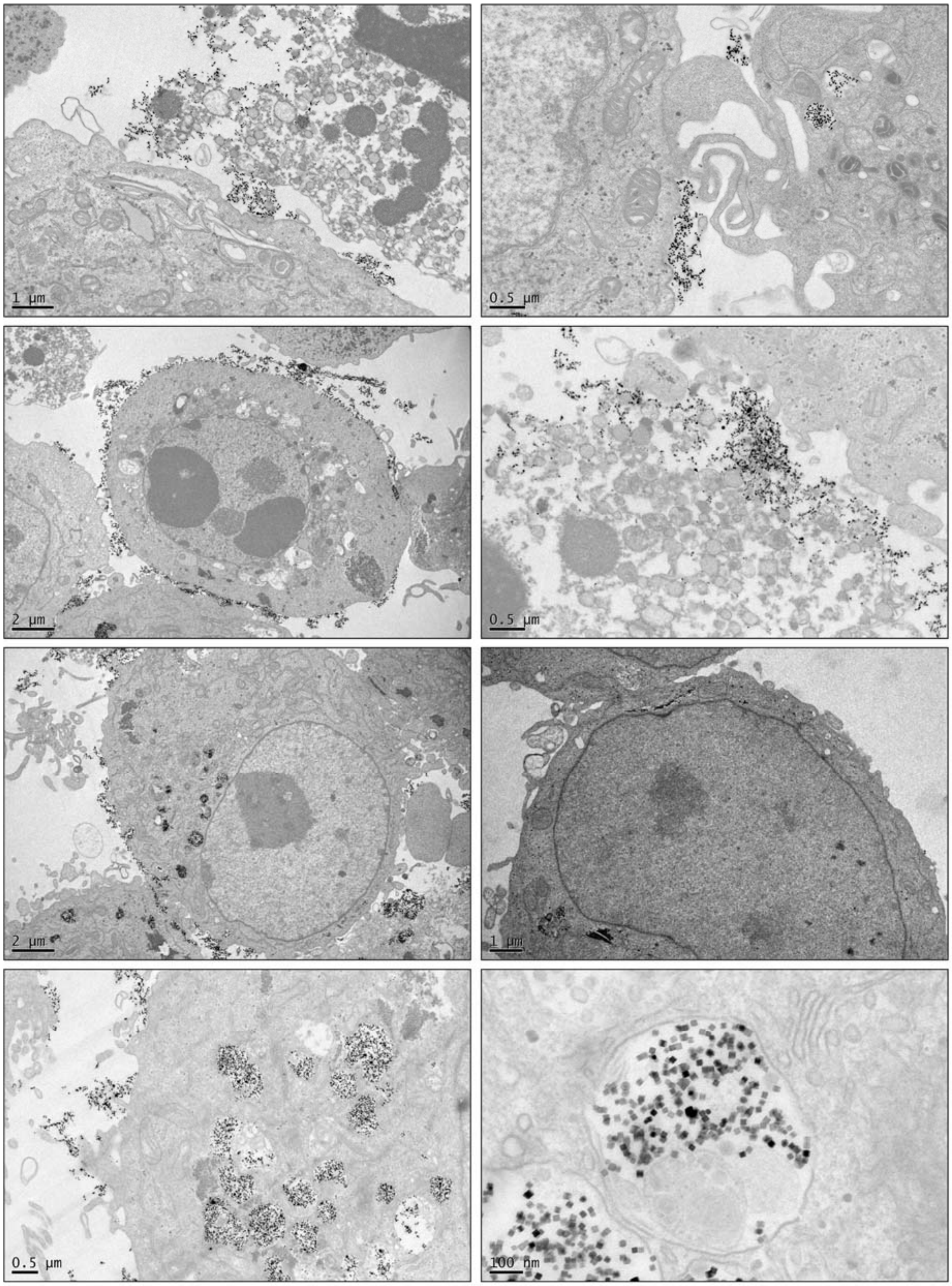

Figure S8. TEM analysis of the CR-CSC #21 cells treated with TR-DOXO for MHT at 45°C during 10 minutes. The cells were processed 1 day post-treatment. The images highlight TR-DOXO internalization while minimal cellular damage is observed.

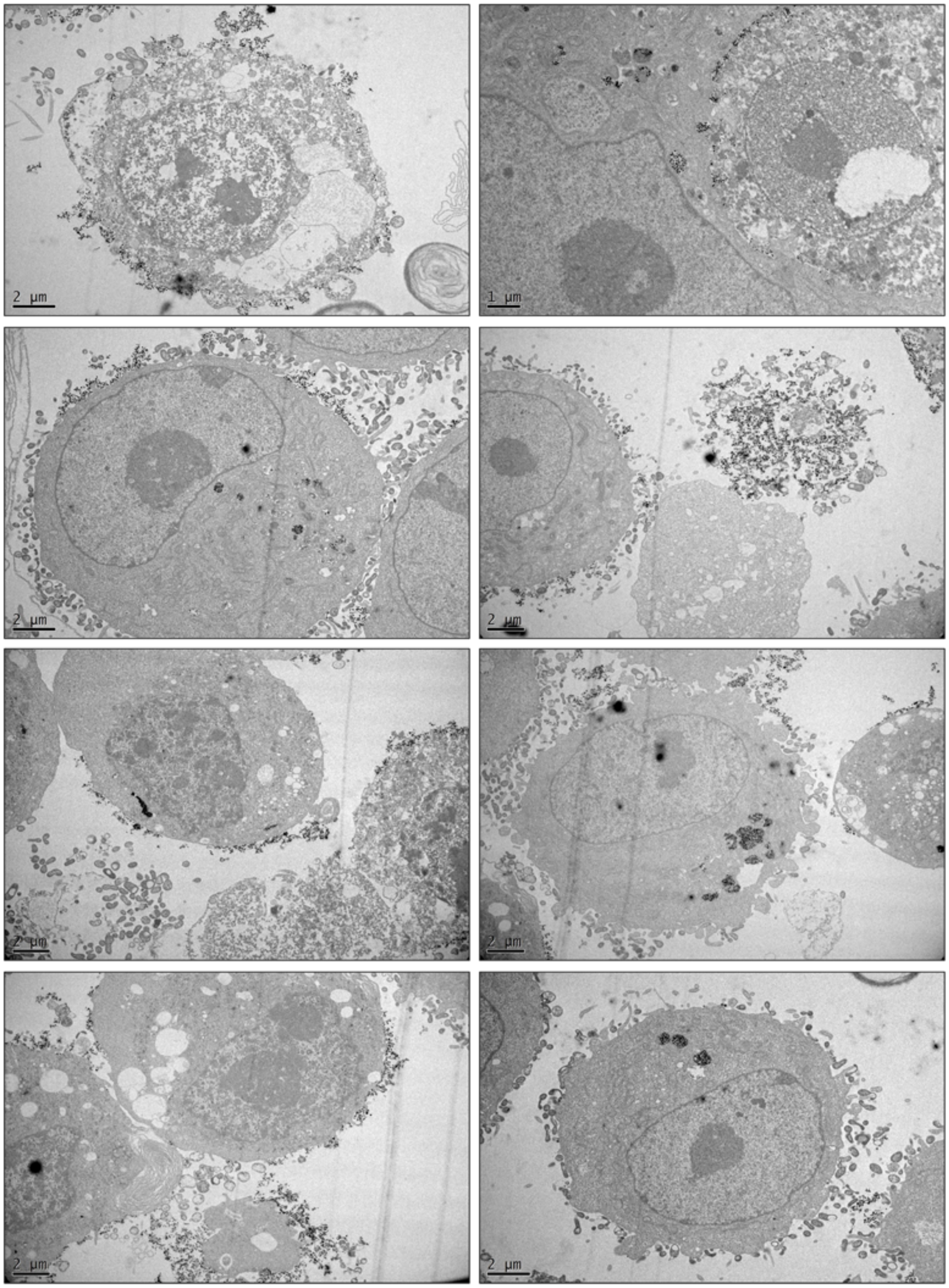

Figure S9. TEM analysis of the CR-CSC #21 treated with TR-DOXO for MHT at 45°C during 30 minutes. The cells were processed 1 day post-treatment. The images highlight TR-DOXO internalization while mild cellular damage is observed.

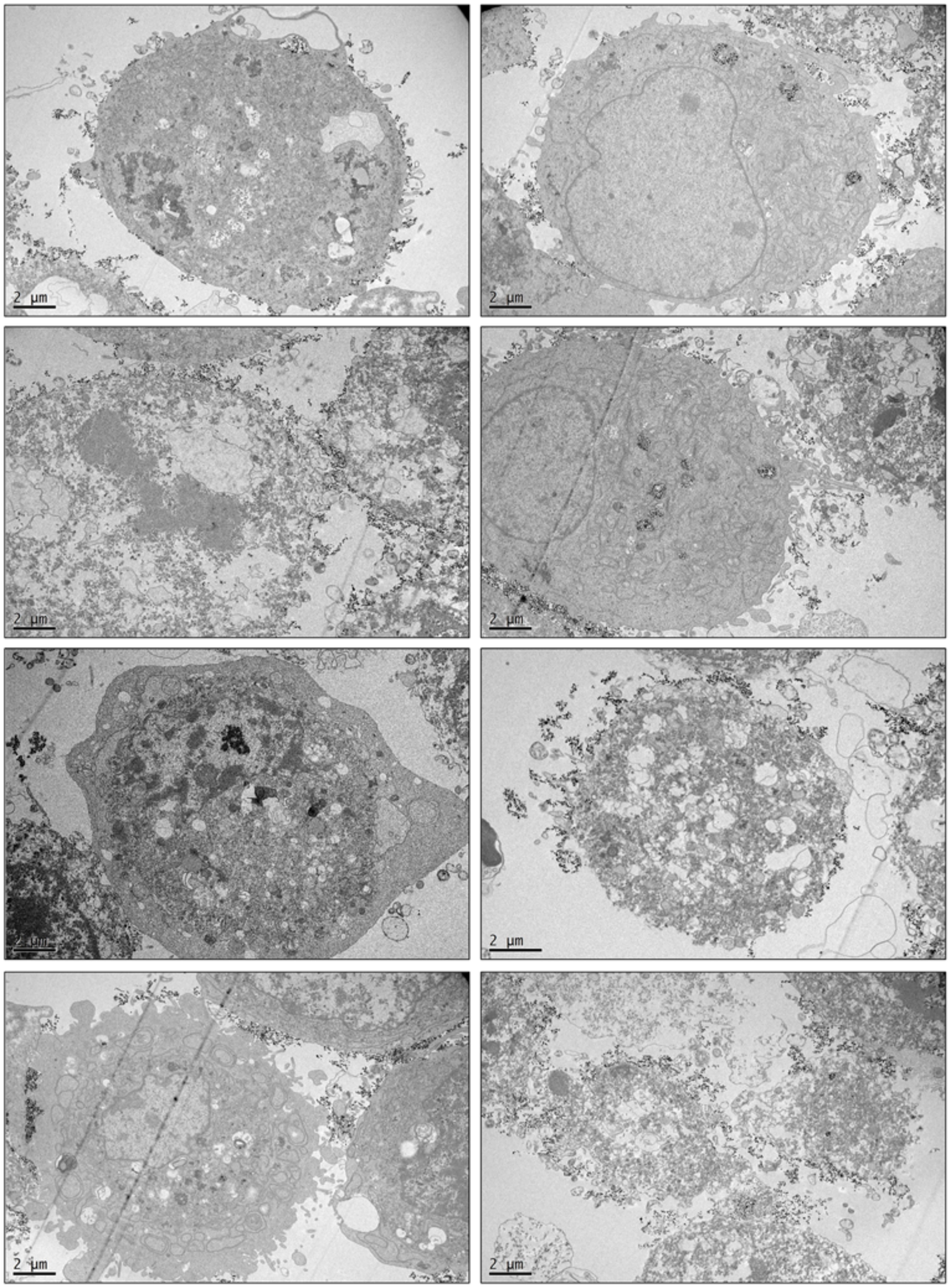

Figure S10 TEM analysis of the CR-CSC #21 cells treated with TR-DOXO for MHT at 45°C during 60 minutes. The cells were processed 1 day post-treatment. The images highlight TR-DOXO internalization and significant cellular damage is observed.

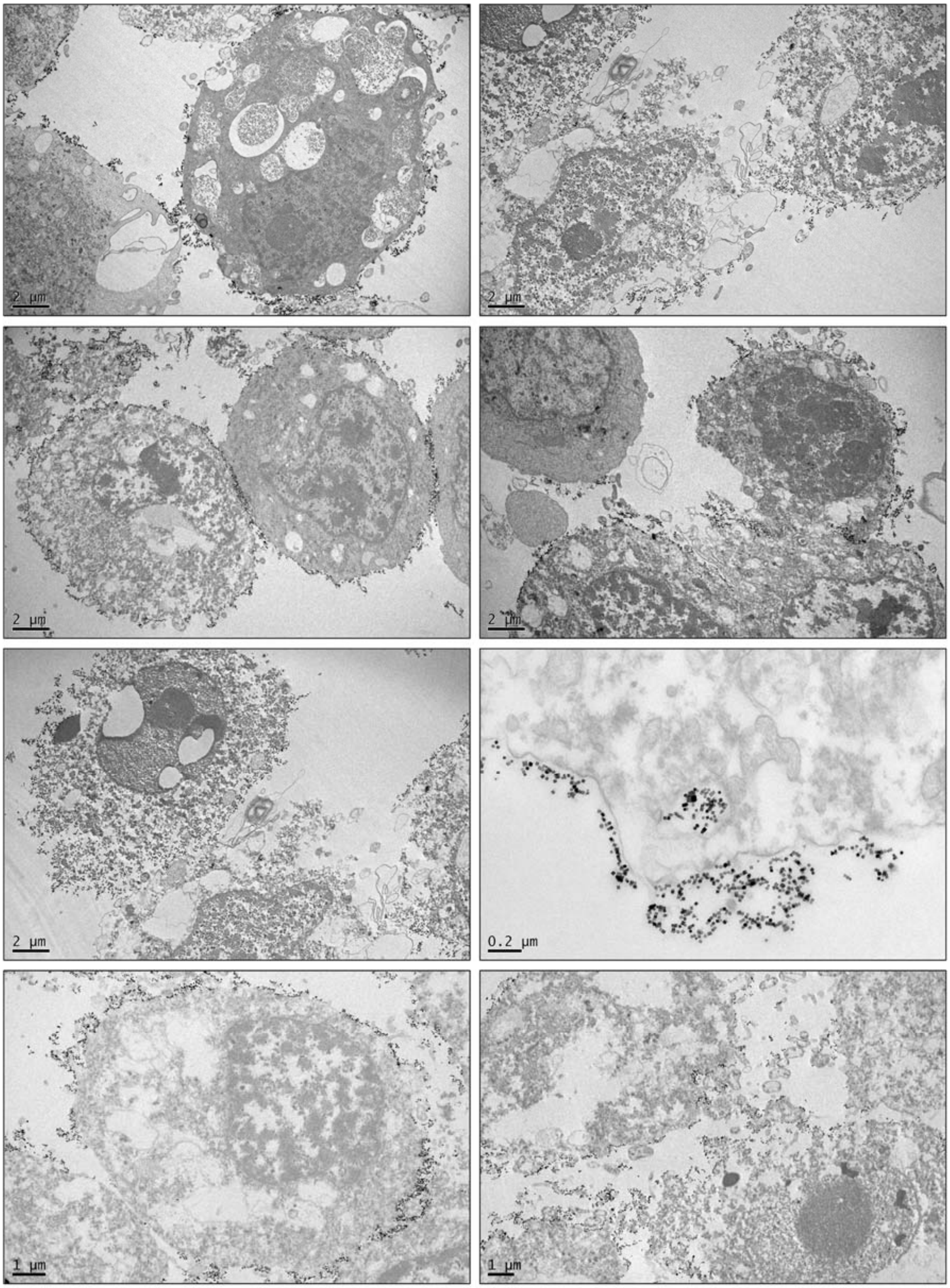

Figure S11 TEM analysis of the CR-CSC #21 cells treated with TR-DOXO for MHT at 45ºC during 90 minutes. The cells were processed 1 day post-treatment. The images highlight low TR-DOXO internalization due to severe cellular damage.

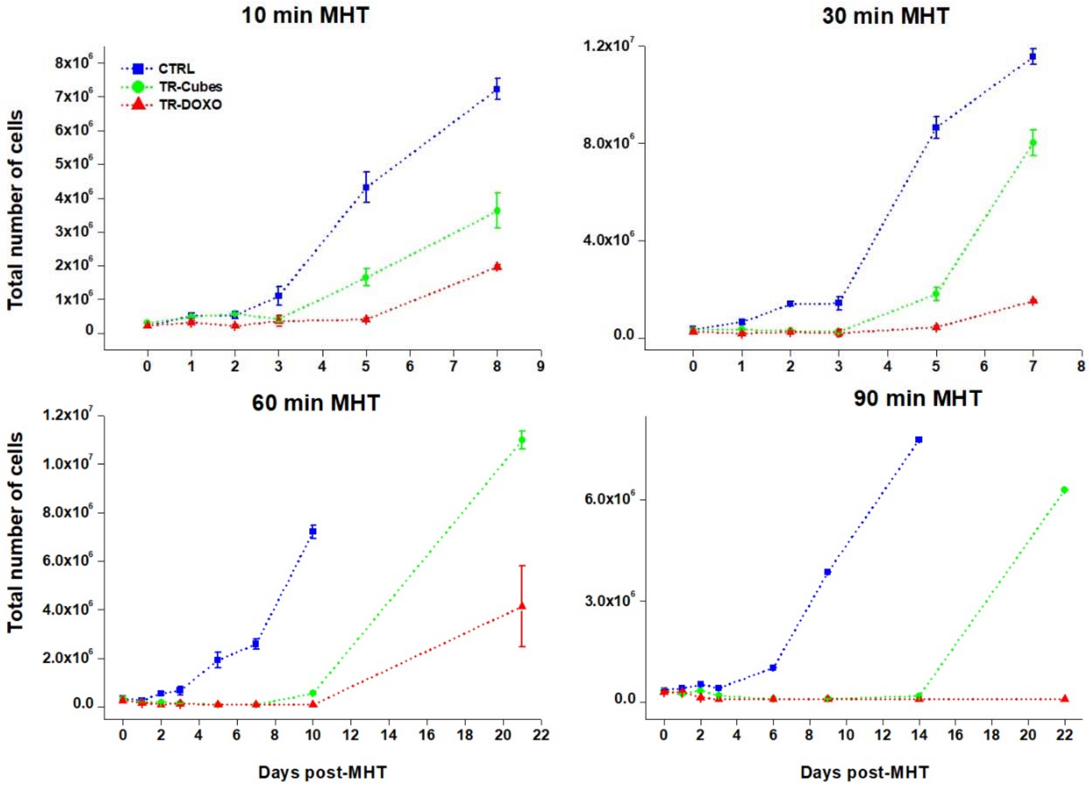

Figure S12. Growth curves of the CR-CSC #21 patient cancer cells treated with magnetic TR-Cubes or TR-DOXO after different time of MHT (10-90 minutes). The blue lines indicate the control cells incubated at 37°C, the green lines the cells treated with TR-Cubes and the red lines the cells treated with TR-DOXO.

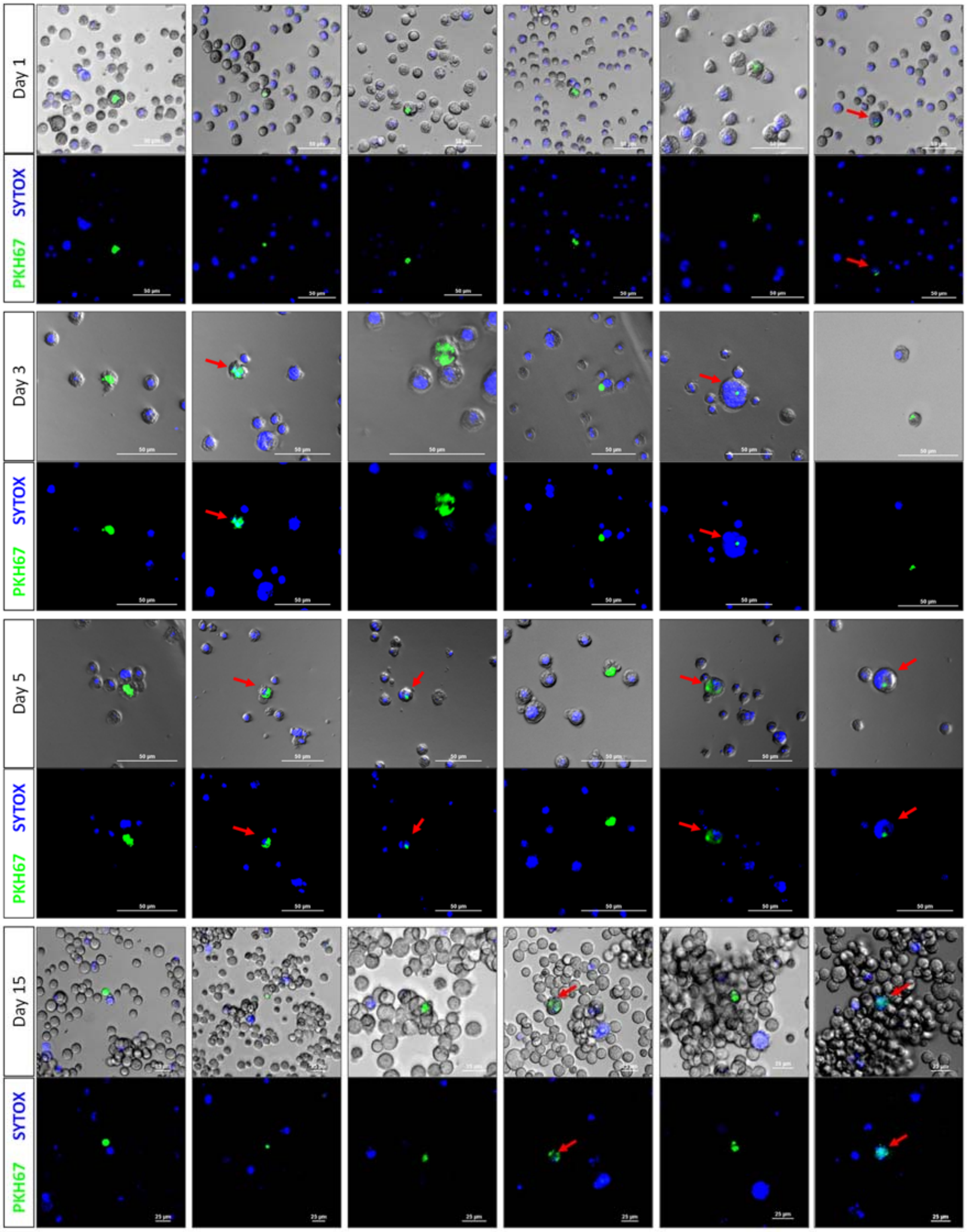

Figure S13. Confocal images of CR-CSC #21 treated with TR-Cubes after MHT during 90 minutes at 45ºC. The pictures show PKH^{pos} quiescent cells death using sytox blue staining, specific for dead cells. The images were acquired over a period of 15 days after the treatment was applied.The green signal represents quiescent PKH^{pos} cells; the blue signal represents dead cells. Scale bars for the images of days 1, 3 and 5 are 50 µm while for day 15 scale bars are of 25 µm. the red arrows indicate colocalization of blue and green signal indicating PKH^{pos} dead cells.

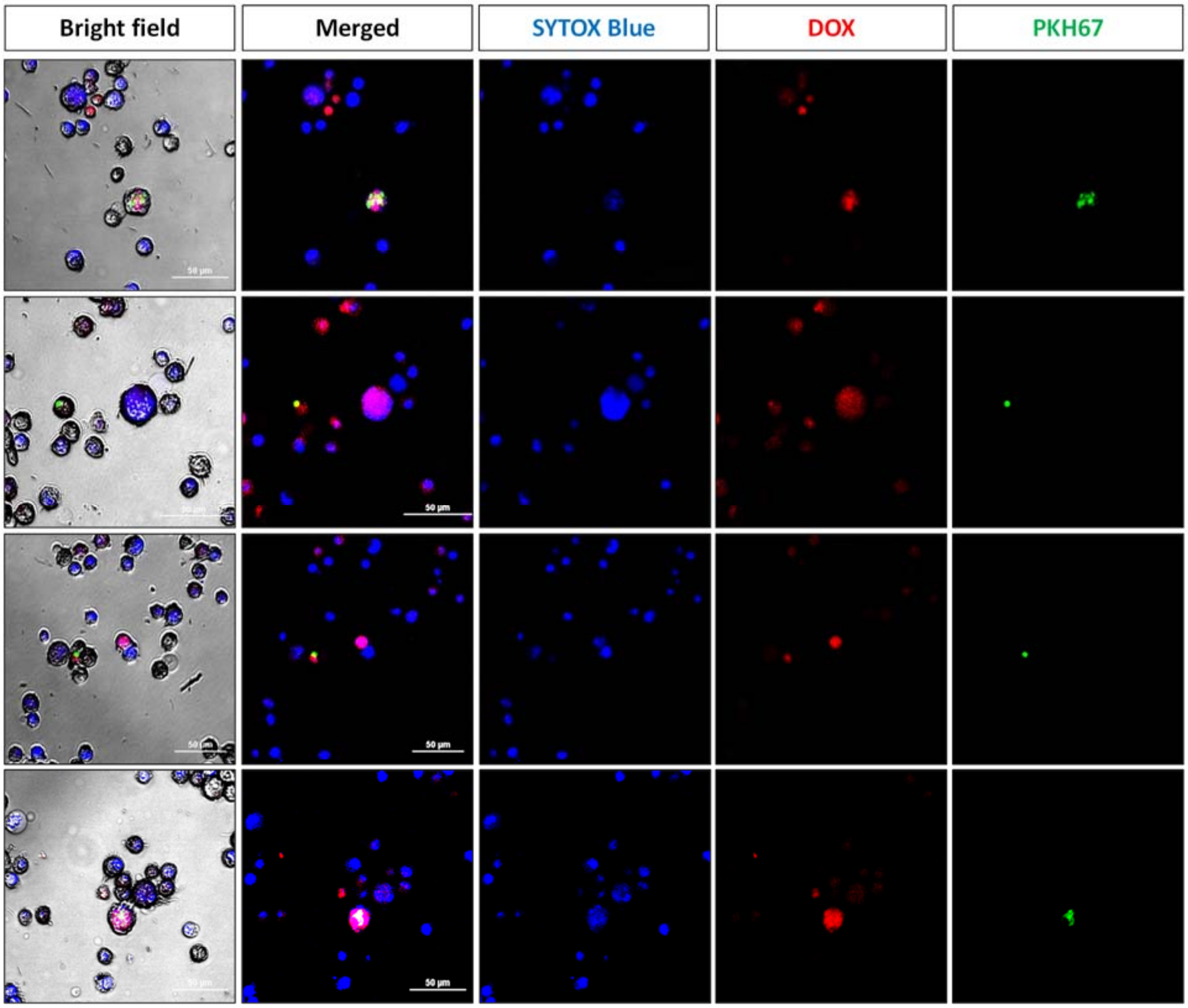

Figure S14. Confocal images of CR-CSC #21 treated with TR-DOXO for PKH^{pos} cell death using sytox blue staining, specific for dead cells. The images were acquired 1 day after the treatment was applied.The green signal represents quiescent PKH^{pos} cells; the blue signal represents dead cells; the red signal represents DOXO. All scale bars are 50 µm.

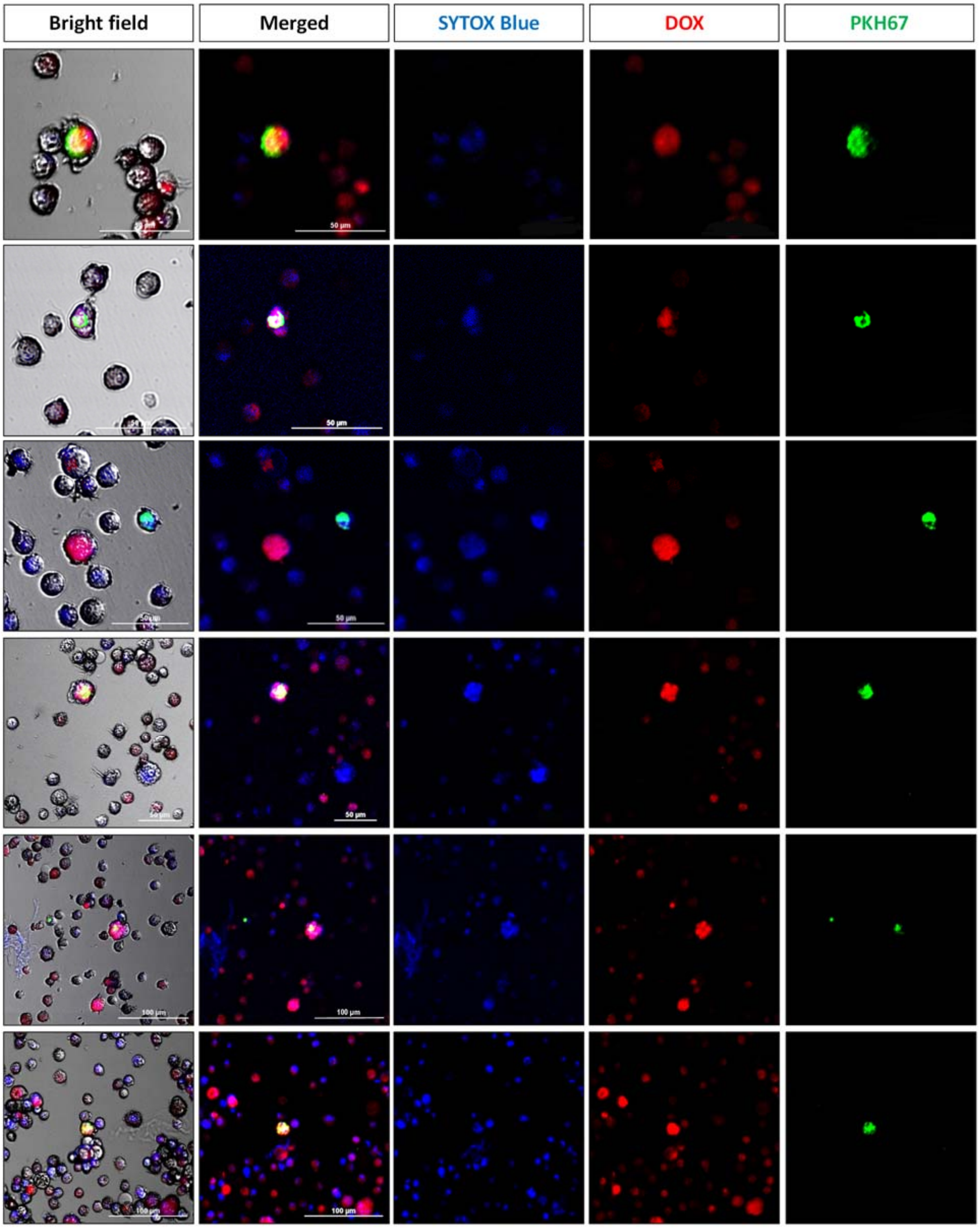

Figure S15. Confocal images of CR-CSC #21 treated with TR-DOXO for PKH^{pos} cell death using sytox blue staining, specific for dead cells. The images were acquired 2 days after the treatment was applied. The green signal represents quiescent PKH^{pos} cells; the blue signal represents dead cells; the red signal represents DOXO. Scale bars are 50 µm for the first four rows and 100 µm for the last two.

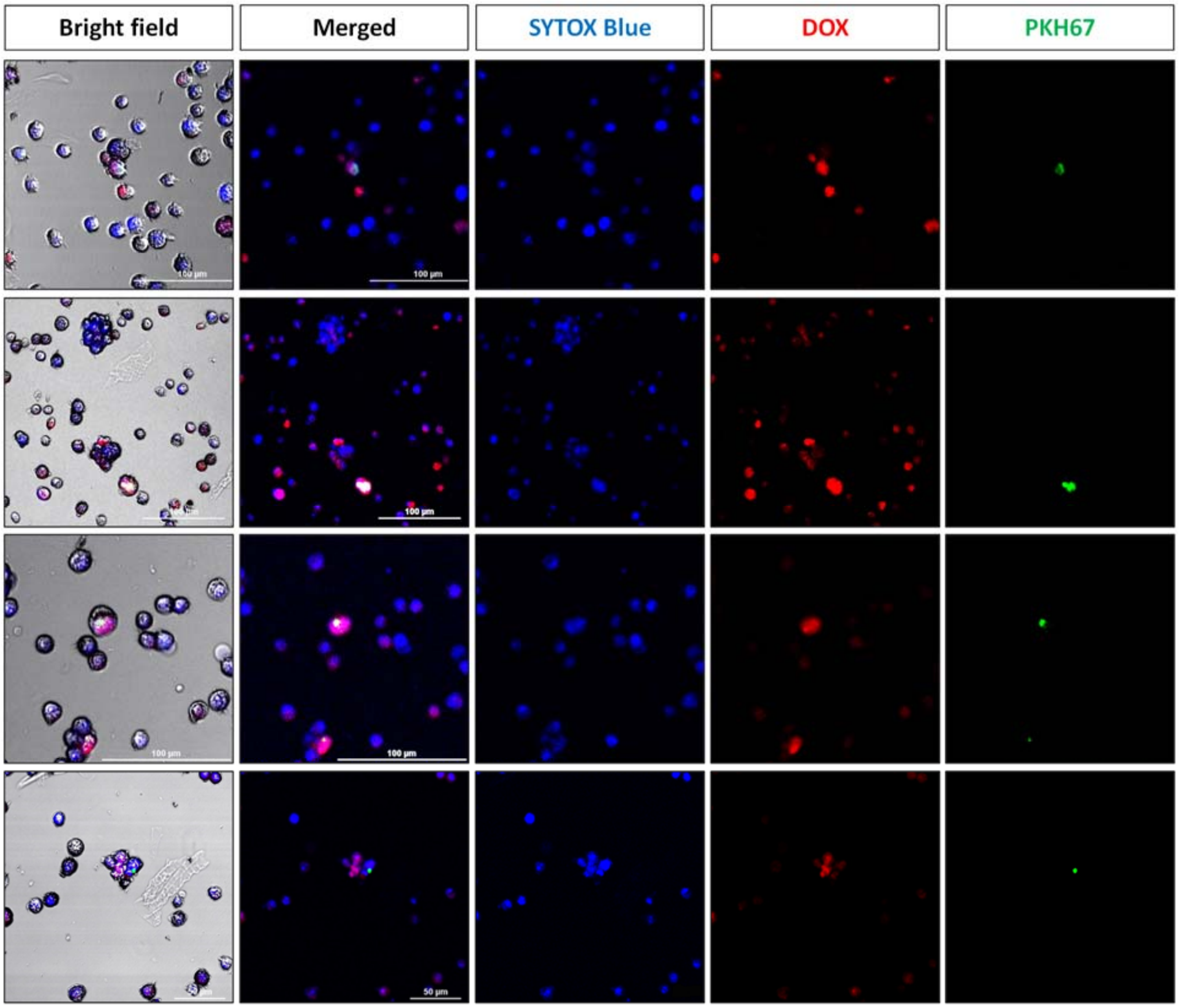

Figure S16. Confocal images of CR-CSC #21 treated with TR_DOXO for PKH^{pos} cell death using sytox blue staining, specific for dead cells. The images were acquired 5 days after the treatment was applied. The green signal represents quiescent PKH^{pos} cells; the blue signal represents dead cells; the red signal represents DOXO. Scale bars are 100 µm except for the last row of pictures in which it is 50 µm.

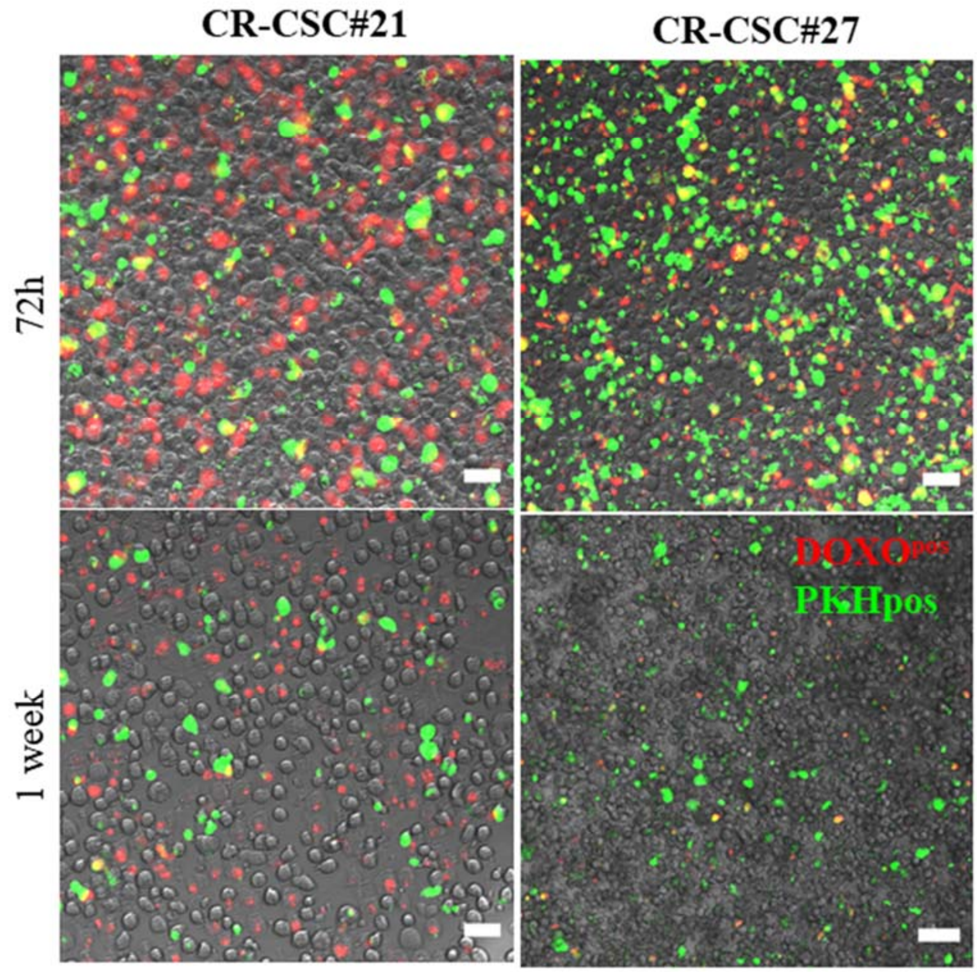

Figure S17. Confocal images of CR-CSC #21 and CR-CSC#27 after 72h and 1 week of DOXO free drug treatment, illustrating almost total absence of DOXO drug (red signal) inside CR-CSC #27 line after 1 week of treatment versus CR-CSC #21. Green signal: PKH^{pos} cells. Scale bar: 50 μm.

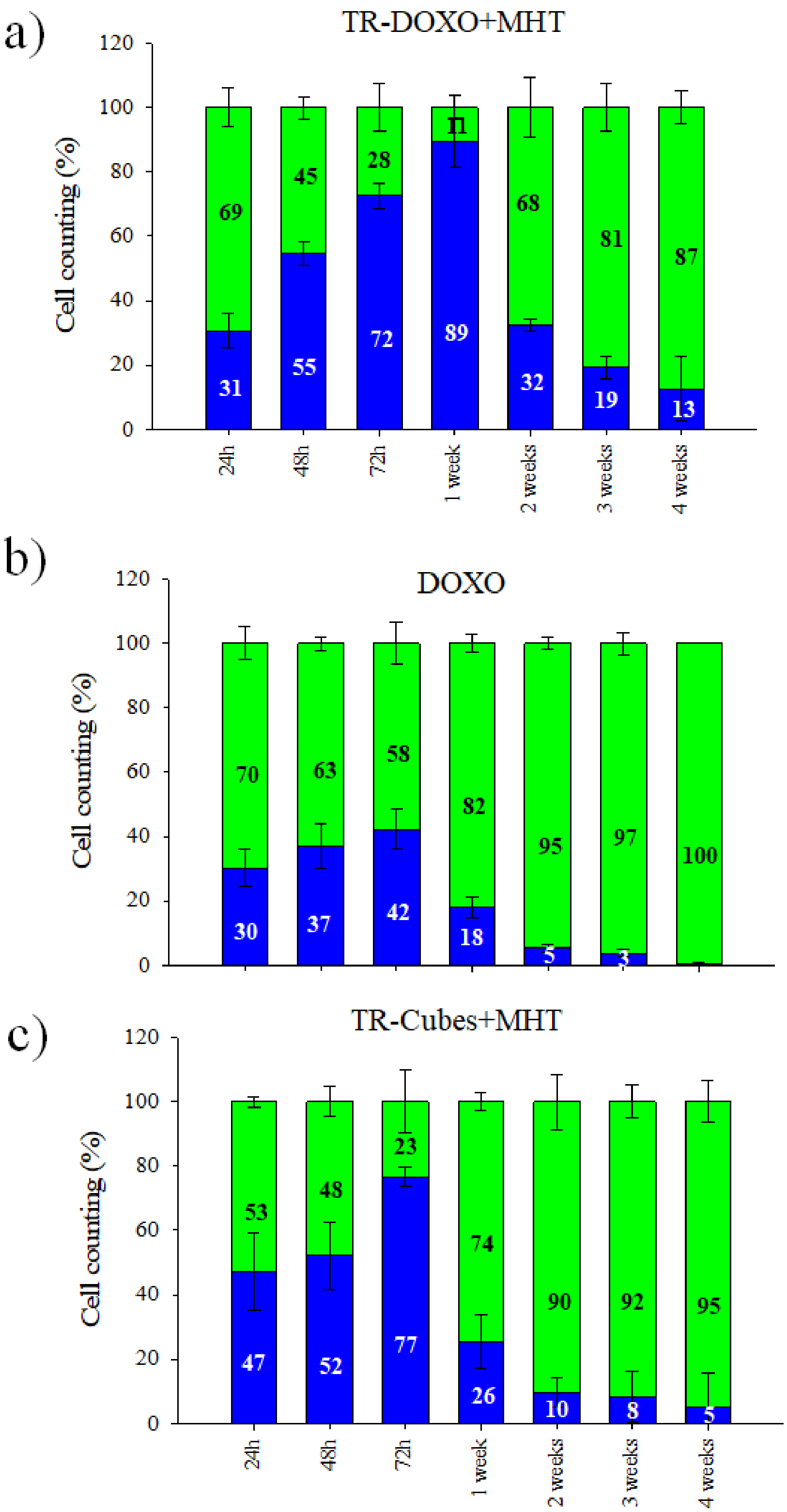

Figure S18. Percentage of CR-CSC #27 CR-CSCs alive (PKH^{pos}/Sytox Blue^{neg}, green bar) or CR-CSC #27 CR-CSCs dead (PKH^{pos} Sytox Blue^{pos}, blue bar) after a) combined treatment with TR-DOXO+MHT,

b) single treatment with DOXO free drug or c) TR-Cubes+MHT treatment. At least 500 cells were counted per each time point and per each experiment in n = 3 independent experimental conditions.

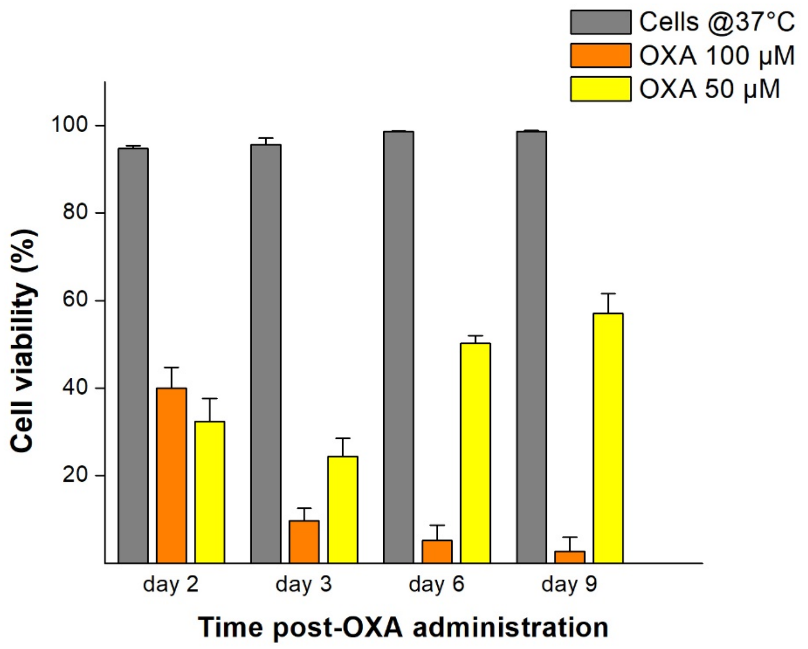

Figure S19 Viability profiles, analysed by trypan blue assay, of CR-CSC #27 after OXA treatment at different concentrations (100 and 50 μM).

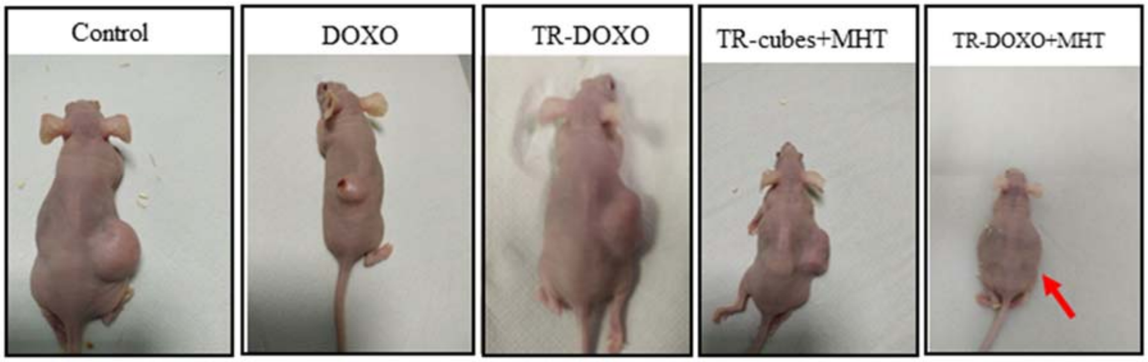

Figure S20. Representative photographs of xenograft colorectal tumors growth for Control animals (no treated CR-CSC #21 cells, 90 days after the treatment), DOXO group (90 days after the treatment), TR-DOXO (60 days after the treatment), TR-Cubes+MHT (100 days after the treatment) and TR-DOXO+MHT (250 days after the treatment) injected cells. Note the absence of tumor in animals injected with the combined and completed treatment TR-DOXO+MHT (red arrow).

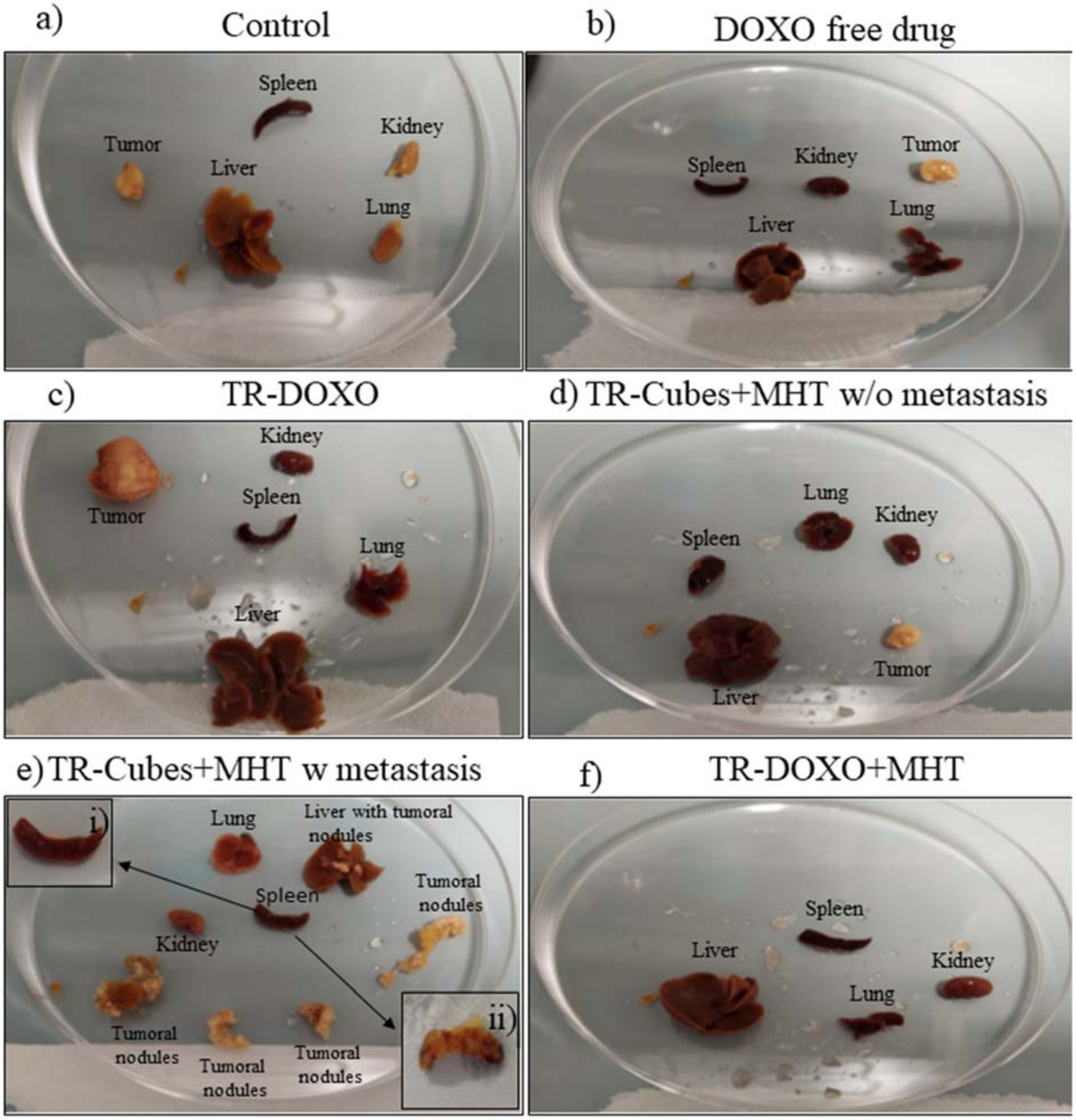

Figure S21. Representative pictures of metastatic tumoral nodules found in animals treated with CR-CSC #21 cells pretreated with TR-cubes+MHT after 150 days of treated cells injection e), in comparison with a) Control, b) DOXO free drug, c) TR-DOXO, d) TR-Cubes+MHT and f) TR-DOXO+MHT treatments. These types of metastasis have been also observed by others. [1] In image e) the insert i) shows some metastatic nodules in the spleen in the insert ii) shows an example of splenomegaly in an animal treated with cells pre-treated with TR-cubes+MHT, 150 days after cells injection, as observed in other studies [2].

[1] Higuchi, T; Yokobori, T; Naito, T; Kakinuma, C; Hagiwara, S; Nishiyama, M; Asao, T., Investigation into Metastatic Processes and the Therapeutic Effects of Gemcitabine on Human Pancreatic Cancer using an Orthotopic SUIT-2 Pancreatic Cancer Mouse Model. *Oncol. Lett.* **2018**, 15, 3091-3099.
[2] Agliano, A; Martin-Padura, I; Marighetti, P; Gregato, G; Calleri, A; Prior, C; Redrado, M; Calvo, A;. Bertolini, F., Therapeutic Effect of Lenalidomide in a Novel Xenograft Mouse Model of Human Blastic NK Cell Lymphoma/Blastic Plasmacytoid Dendritic Cell Neoplasm, *Clin. Cancer. Res.* **2011**, 17, 6163-6173.
